# Icaritin Ameliorates Cisplatin‐Induced Mitochondrial Metabolic Dysfunction‐Associated Nephrotoxicity and Synergistically Potentiates Its Antitumor Efficacy

**DOI:** 10.1002/advs.202506712

**Published:** 2025-11-30

**Authors:** Piao Luo, Junhui Chen, Yehai An, Kun Meng, Wei Zhou, Wenhui Li, Jing Liu, Wentong Zhao, Weiyi He, Ting Cao, Jingnan Huang, Sha Feng, Shiguang Yang, Hongling Hu, Jiaxian Liao, Hengkai He, Mingjing Hao, Qian Zhang, Jigang Wang, Yue Gao

**Affiliations:** ^1^ Guangdong Basic Research center of Excellence for Integrated Traditional and Western Medicine for Qingzhi Diseases Guangdong provincial Key Laboratory of Chinese Medicine pharmaceutics School of Traditional Chinese Medicine and School of pharmaceutical Sciences Southern Medical University Guangzhou Guangdong 510515 China; ^2^ Department of Pharmaceutical Sciences Beijing Institute of Radiation Medicine Beijing 100850 China; ^3^ Department of Pulmonary and Critical Care Medicine Shenzhen Institute of Respiratory Diseases and Shenzhen Clinical Research Centre for Geriatrics Shenzhen People's Hospital First Affiliated Hospital of Southern University of Science and Technology Second Clinical Medical College of Jinan University Shenzhen Guangdong 518020 China; ^4^ State Key Laboratory for Quality Ensurance and Sustainable Use of Dao‐di Herbs Artemisinin Research Center and Institute of Chinese Materia Medica China Academy of Chinese Medical Sciences Beijing 100700 China

**Keywords:** cisplatin, icaritin, mitochondrial dysfunction, multi‐omics, nephrotoxicity

## Abstract

Cisplatin (CDDP) is a highly effective chemotherapy drug with broad clinical utility. Yet its therapeutic application is significantly constrained by off‐target toxicities, especially nephrotoxicity. However, the molecular mechanisms underlying CDDP‐induced kidney injury remain incompletely elucidated. Here, integrated multi‐omics approaches are employed to dissect the pathophysiology of CDDP nephrotoxicity and uncover that CDDP directly binds to mitochondrial proteins, causing metabolic dysfunction and impairing mitochondrial respiration. Additionally, CDDP triggers mitochondrial reactive oxygen species generation, activating the nuclear factor kappa‐B (NF‐κB) signaling pathway and downstream inflammatory effectors. scRNA‐seq analysis reveals remarkable cellular heterogeneity in the renal response to CDDP exposure. Mechanistically, it is identified that CDDP‐bound proteins are predominantly localized in proximal tubular (PT) cells. Ligand–receptor analysis demonstrates that CDDP‐damaged PT cells recruit and activate renal immune cells in tumor‐bearing mice, exacerbating renal injury. Notably, icaritin (ICA) effectively mitigates CDDP‐induced reactive oxygen species (ROS) accumulation, suppresses NF‐κB activation and inflammation, and restores metabolic homeostasis. Combinatorial treatment with ICA not only ameliorates CDDP‐induced nephrotoxicity but also enhances its anti‐cancer efficacy. Taken together, these findings provide novel mechanistic insights into CDDP nephrotoxicity and propose a dual‐function therapeutic strategy to optimize CDDP‐based cancer therapy while minimizing renal damage.

## Graphical abstract

In this study, scRNA‐seq and chemoproteomics are integrated to characterize CDDP‐bound proteins at single‐cell resolution in tumor‐bearing mice. Additionally, the research demonstrates that ICA alleviates CDDP‐induced nephrotoxicity while enhancing its chemotherapeutic efficacy. These findings not only deepen the mechanistic understanding of CDDP‐induced nephrotoxicity but also establish a theoretical basis for the clinical translation of CDDP‐based combination therapeutic regimens.

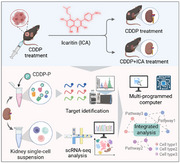

## 1 Introduction

CDDP, a highly effective chemotherapy agent, is widely used in the treatment of various malignancies.^[^ ^1^, ^2^ ^]^ The CDDP requires intracellular bioactivation, wherein chlorides are substituted by water molecules.^[^ ^3^ ^]^ This activation process generates highly reactive species binding to DNA, inducing cytotoxic lesions within tumors.^[^ ^4^ ^]^ However, the undesirable accumulation of CDDP may also provoke cytotoxic effects in healthy tissues. Indeed, the CDDP clinical application is constrained by tissue toxicities, particularly nephrotoxicity.^[^ ^5^ ^]^ CDDP predominantly induces injury and death of proximal tubular cells through mechanisms such as apoptosis and mitochondrial dysfunction.^[^ ^6^, ^7^ ^]^ Besides DNA damage, a positive feedback loop involving the inflammatory response and oxidative stress has been identified as a significant mechanism underlying CDDP‐induced nephrotoxicity.^[^ ^8^ ^]^ Although CDDP is recognized for its ability to bind to DNA and cause cellular damage, it is still uncertain whether CDDP directly interacts with proteins to induce renal toxicity. Therefore, the underlying mechanisms of CDDP‐induced nephrotoxicity require further exploration to inform therapeutic strategies.

Recent advances in multi‐omics integration strategies have been extensively utilized in pharmacological and toxicological research, enabling systematic, comprehensive, and accurate elucidation of drug action mechanisms.^[^ ^9^, ^10^, ^11^ ^]^ Single‐cell RNA sequencing (scRNA‐seq) technology has rapidly evolved to elucidate renal cellular identities and dynamic states during injury and therapeutic intervention.^[^ ^12^, ^13^, ^14^ ^]^ Concurrently, chemoproteomics is considered as a powerful tool for direct target discovery of bioactive molecules, significantly advancing mechanistic insights and drug development.^[^ ^15^, ^16^, ^17^, ^18^ ^]^ Based on these methodologies, single‐cell target profiling (STEP) is developed to integrate scRNA‐seq and chemoproteomics datasets.^[^ ^19^ ^]^ Previously, we employed a combination of chemoproteomics, scRNA‐seq, and STEP to elucidate the direct targets and cellular heterogeneity in aristolochic acid‐induced hepatorenal toxicity.^[^ ^19^, ^20^, ^21^, ^22^ ^]^ This approach is poised to unveil the global landscape of CDDP‐bound proteins in the kidneys of tumor‐bearing mice at single‐cell resolution, thereby providing a theoretical foundation for developing nephroprotection strategies and synergistic therapeutic regimens.

Despite preclinical studies identifying potential drug candidates that mitigate CDDP‐induced nephrotoxicity while maintaining its antitumor efficacy,^[^ ^1^, ^23^, ^24^ ^]^ clinical prevention of CDDP nephrotoxicity still relies predominantly on nonspecific interventions. Notably, up to 35% of patients experience CDDP‐induced nephrotoxicity, often requiring dose reduction or treatment discontinuation, which significantly compromises patient outcomes.^[^ ^25^ ^]^ This underscores an unmet clinical urgency for strategies that mitigate CDDP‐induced nephrotoxicity while preserving its oncolytic efficacy. Icaritin (ICA), a bioactive flavonoid isolated from the aerial parts of *Epimedium* species, exhibits well‐characterized antioxidant, anti‐inflammatory, hepatorenal protective, and anticancer properties.^[^ ^26^, ^27^, ^28^ ^]^ Approved as a Class 1.2 innovative traditional Chinese medicine drug in China, ICA capsules have been established as a therapeutic option for advanced hepatocellular carcinoma (HCC). However, whether ICA can mitigate CDDP‐induced nephrotoxicity while potentiating its antitumor efficacy in tumor‐bearing mouse models remains entirely uninvestigated.

Here, we integrated scRNA‐seq, chemoproteomics, and STEP technologies pipeline to characterize CDDP‐bound proteins at single‐cell resolution in tumor‐bearing mice with treated CDDP. Our results reveal that CDDP directly binds to mitochondrial proteins, inducing mitochondrial reactive oxygen species (MitoROS) production. This triggers nuclear translocation of NF‐κB transcription factors, activating an inflammatory cascade that culminates in proximal tubular (PT) cell damage. Importantly, we demonstrate that ICA mitigates CDDP‐induced nephrotoxicity while potentiating its chemotherapeutic efficacy. These findings not only enhance our mechanistic understanding of CDDP‐induced nephrotoxicity but also lay a theoretical foundation for the clinical translation of CDDP‐based combination regimens.

## 2 Results

### 2.1 Cisplatin Induces Nephrotoxicity in Tumor‐Bearing Mice

**Figure 1:**
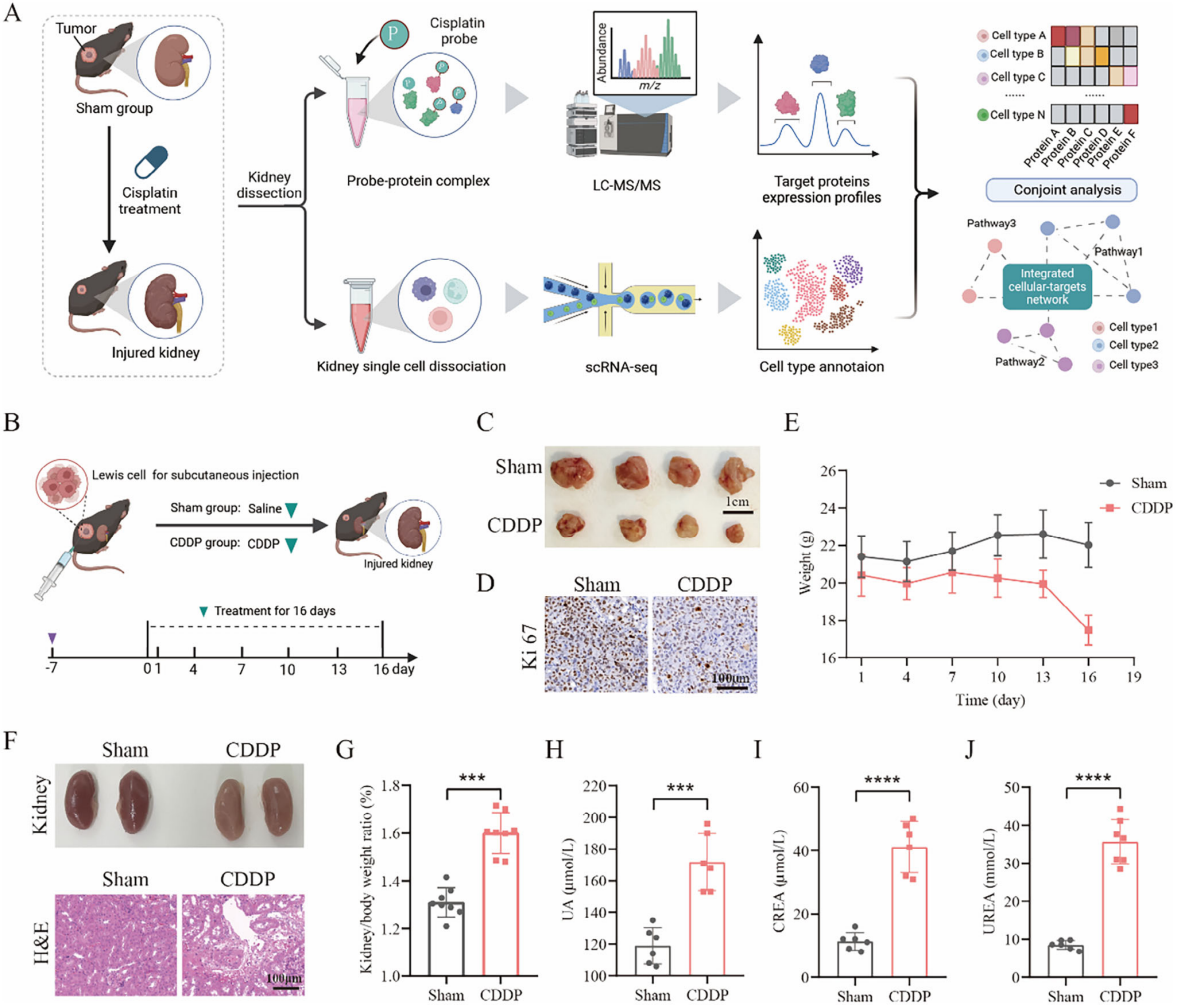
Cisplatin exerts chemotherapeutic effects and induces nephrotoxicity. A) The experimental flow planning diagram presented in this paper. B) Protocol for conducting animal experiments aimed at evaluating the therapeutic efficacy and nephrotoxicity of CDDP in the treatment of tumors. C) Representative tumor morphology in Sham and CDDP groups after 16 days of treatment (scale bar = 1 cm). D) Immunohistochemical assay for Ki67 marker in both groups (scale bar = 100 µm). E) Body weight statistics in both groups (*n* = 8–9). F) Representative kidney morphology, and H&E staining results of renal tissues in both groups (scale bar = 100 µm). G) The kidney/body weight ratio in both groups (*n* = 8–9). H–J) Effects of CDDP on UA, CREA, and UREA in both groups (*n* = 6). The error bars indicate the means ± SD. The *p‐*values were determined by Student's *t*‐test; ***p < 0.001, ****p < 0.0001 versus Sham.

To systematically evaluate the molecular nephrotoxicity profile in cisplatin‐treated renal cell populations, we performed scRNA‐seq and chemoproteomics on wild‐type (Sham) and cisplatin‐treated (CDDP) kidney tissues from tumor‐bearing mice with or without CDDP treatment (**Figure** **1**A,B). First, to validate the reliability of the model established by administering CDDP, we conducted preliminary experiments to optimize the treatment protocol (Figure S1A–C, Supporting Information). The CDDP‐10 mg kg^−1^ group has been demonstrated to effectively inhibit tumor growth (Figure 1C; Figure S1D,E, Supporting Information), then reduce tumor cell proliferation (Figure 1D; Figure S1F, Supporting Information). However, its administration has been associated with mild weight loss in mice (<20%) (Figure 1E). From the macroscopic appearance and pathological staining of the kidneys, it was observed that compared with the Sham group, the kidneys in the CDDP group showed significant pathological damage (Figure 1F; Figure S1G, Supporting Information). Furthermore, our data indicate that CDDP significantly elevated the renal‐to‐body ratio (Figure 1G) and resulted in increased blood biochemical markers, specifically uric acid (UA), creatinine (CREA), and urea (UREA) (Figure 1H–J), suggesting that CDDP induces significant nephrotoxicity. Simultaneously, it also results in an increase in the liver‐to‐body ratio and elevated blood levels of the liver biochemical markers (Figure S1H–J, Supporting Information), indicating that CDDP induces a certain degree of liver injury. In summary, while CDDP demonstrates potent anticancer efficacy, it is concomitantly associated with substantial side effects, with renal toxicity being particularly prominent.

### 2.2 Cisplatin Induces Renal Toxicity by Binding to Mitochondrial Proteins

**Figure 2:**
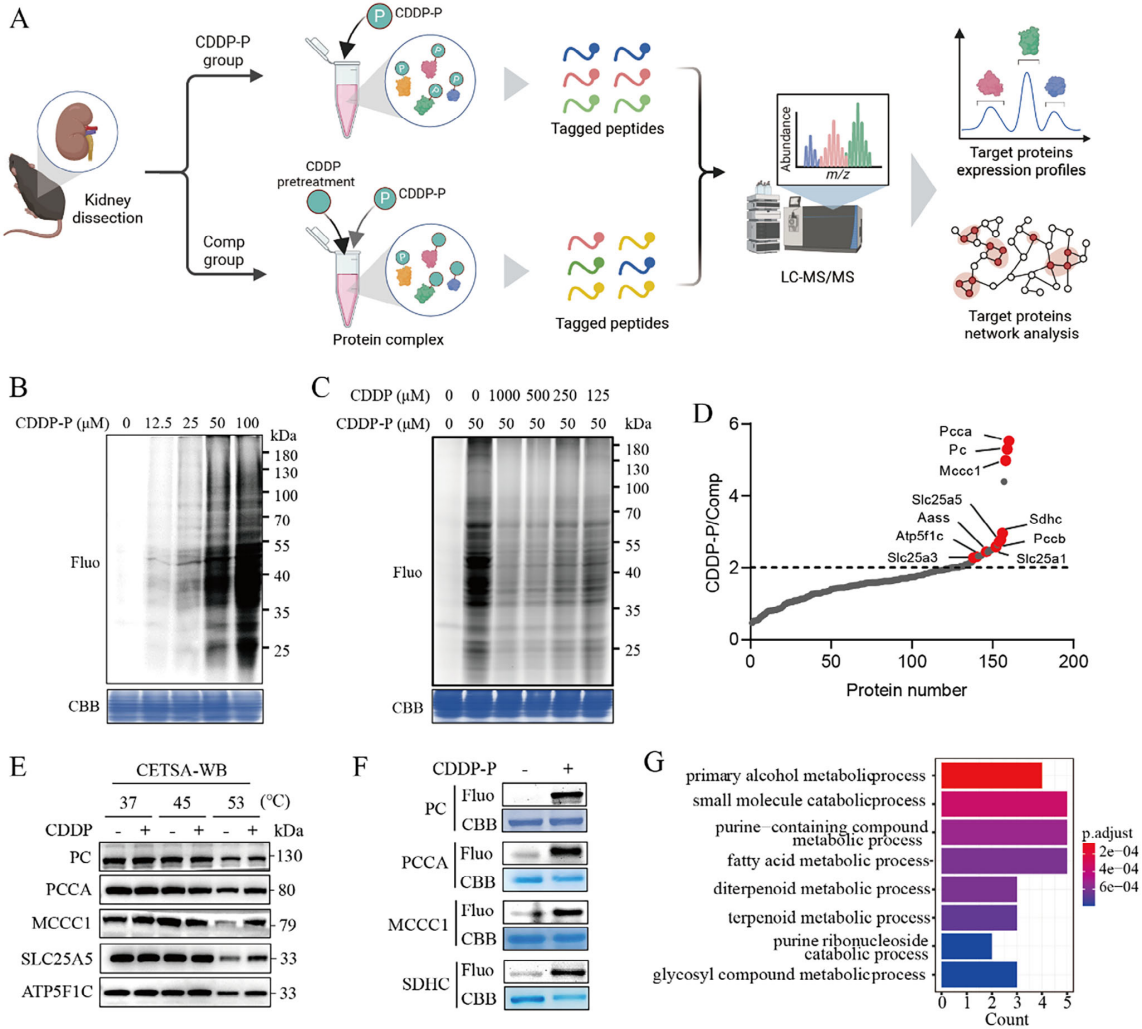
Chemoproteomics reveals that cisplatin induces renal toxicity by targeting mitochondrial proteins. A) Overall workflow for the chemoproteomics profiling of potential CDDP‐acting targets in the kidney. B) Protein labeling with CDDP‐P in kidney lysis. C) Proteins with labeled CDDP‐P and CDDP. D) CDDP targeting mitochondrial proteins; Comp group, pre‐incubation with excess CDDP to compete with CDDP‐P bound proteins. E) CETSA‐WB assay to detect CDDP‐bound mitochondrial proteins. F) Fluorescent labeling assay to access CDDP‐bound residues of recombinant PC (Pro36 ~ Glu486), PCCA (Val580 ~ Glu728), MCCC1 (Ala512 ~ Glu725), and SDHC (Met1 ~ Arg72) proteins. G) CDDP‐binding proteins involving in metabolic processes.

CDDP has been shown to directly bind to DNA, inducing DNA damage and exerting its anticancer activity.^[^ ^29^ ^]^ Paradoxically, this same DNA‐binding mechanism contributes to its multi‐organ toxicity. Notably, whether CDDP directly interacts with target proteins remains poorly defined. Therefore, we aim to employ chemoproteomics to identify CDDP binding to protein targets, thereby illuminating the mechanistic basis of its nephrotoxic effects. To identify potential targets of CDDP, we synthesized a CDDP probe (CDDP‐P) conjugated with an alkyne tag^[^ ^30^ ^]^ (Figure S2A,B, Supporting Information). Subsequently, following chemoproteomics methodology established in prior studies,^[^ ^18^, ^31^ ^]^ we performed target identification assays to characterize CDDP's direct protein interactors (**Figure** **2**A). Using a labeling assay in kidney lysates, we observed that CDDP‐P bound to specific proteins (Figure 2B). Additionally, co‐incubation with excess CDDP can compete with CDDP‐P‐bound proteins in kidney lysates (Figure 2C). Using liquid chromatography‐tandem mass spectrometry (LC/MS/MS) for CDDP‐bound protein identification, comparative analysis of differentially expressed proteins between CDDP‐P and Comp groups revealed that CDDP preferentially interacts with mitochondrial proteins, including propionyl‐CoA carboxylase subunit alpha (PCCA), pyruvate carboxylase (PC), ATP synthase F1 subunit gamma (ATP5F1C), solute carrier family 25 member 5 (SLC25A5), and succinate dehydrogenase complex subunit C (SDHC) (Figure 2D). Bioinformatics enrichment analysis identified significant involvement of these mitochondrial proteins in metabolic pathways (Figure 2G). Using cellular thermal shift assay (CETSA) coupled with Western blot (WB), we further demonstrated that CDDP significantly enhanced the thermal stability of mitochondrial proteins PC, PCCA, SLC25A5, ATP5F1C, and methylcrotonyl‐CoA carboxylase subunit 1 (MCCC1) (Figure 2E). Concurrently, molecular docking predictions revealed that CDDP binds to the crucial regions of PC, PCCA, SLC25A5, MCCC1, and SDHC, leading to disrupted ATP production (Figure S2C,D, Supporting Information). Furthermore, we utilized the crucial domains of recombinant PC, PCCA, MCCC1, and SDHC proteins to conduct fluorescence labeling experiments. These experiments demonstrated the presence of strong fluorescent signals in the complexes that form between CDDP‐P and the aforementioned protein domains (Figure 2F). Collectively, these findings indicate that CDDP directly interacts with the crucial domains of these mitochondrial proteins.

### 2.3 Single Cell Transcriptomic Profiling Reveals Significant Cellular Heterogeneity upon Cisplatin Exposure

**Figure 3:**
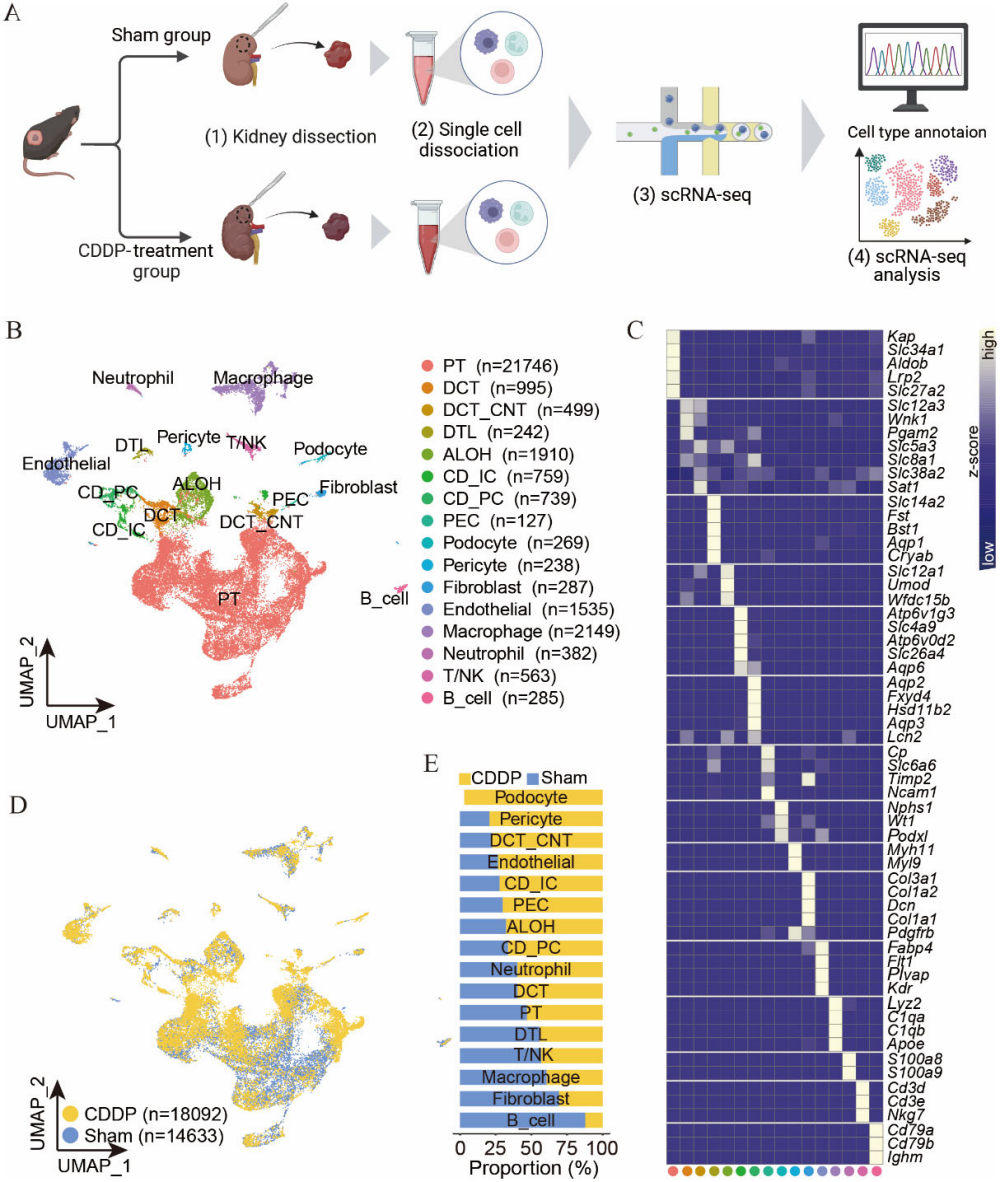
Cisplatin reprograms the transcriptome of the kidney in the tumor mouse. A) Experimental procedure of scRNA‐seq in the kidney of a tumor mouse with or without CDDP treatment. B) UMAP track displays cell types on single‐cell transcriptomes. C) Heat map shows the top marker genes in each cell type. D,E) Cellular proportion of cell types in Sham and CDDP groups. PT, proximal tubule; DCT, distal convoluted tubule; CNT, connecting tubule; DTL, descending limb of loop of Henle; ALOH, thin ascending limb of loop of Henle; CD_ID, intercalated cells of collecting duct; CD_PC, principal cells of collecting duct; PEC, parietal epithelial cells.

Although it has been established that CDDP directly binds to mitochondria‐associated proteins, its effects on the renal microenvironment and cellular heterogeneity remain unclear. We employed the scRNA‐seq technique similar to that utilized in our previous studies^[^ ^21^, ^32^, ^33^ ^]^ to elucidate the effects of CDDP on microenvironment and cellular heterogeneity in the kidneys from the control (Sham) and CDDP‐treated group (CDDP) (**Figure** **3**A). We obtained both the Sham and CDDP group single‐cell datasets, and processed them to reveal CDDP‐treated kidney cellular atlases. After quality control and filtering, we identified 32 725 high‐quality cells from 18 092 CDDP‐treated cells and 14 633 Sham cells (Figure 3D; Figure S3A–C, Supporting Information). We annotated the different cell types with gene expression markers: *Lrp2^+^Slc27a2^+^* PTs, *Slc12a3^+^Slc8a1^+^* DCT cells and *Sat1^+^* DCT_CNT cells, *Aqp1^+^Bst1^+^* DTL cells, *Slc12a1^+^Umod^+^* ALOH, *Atp6v1g3^+^Atp6v0d2^+^* CD‐IC cells, and *Aqp2^+^Fxyd4^+^* CD‐PC cells, *Ncam1^+^* PEC cells, *Nphs1^+^Wt1^+^* podocyte cells, *Myh11^+^* pericyte cells, *Col1a2^+^Dcn^+^* Fibroblast, *Kdr^+^Flt1^+^* endothelial cells, *Lyz2^+^* macrophages, *S100a8^+^* Neutrophil cells, *Cd3d^+^Nkg7^+^* T/NK and *Cd79a^+^Ighm^+^* B cells (Figure 3B,C; Figure S3D,E, Supporting Information). Consequently, the majority of nephron cells and endothelial cells were predominantly derived from the CDDP‐treated samples, while immune cells were primarily derived from the Sham samples (Figure 3E). Collectively, the mouse kidney single‐cell datasets revealed the heterogeneous transcriptome microenvironment between Sham and CDDP‐treated group cells.

### 2.4 Cisplatin Selectively Deactivates Biological Pathways in Proximal Tubular Cells

**Figure 4:**
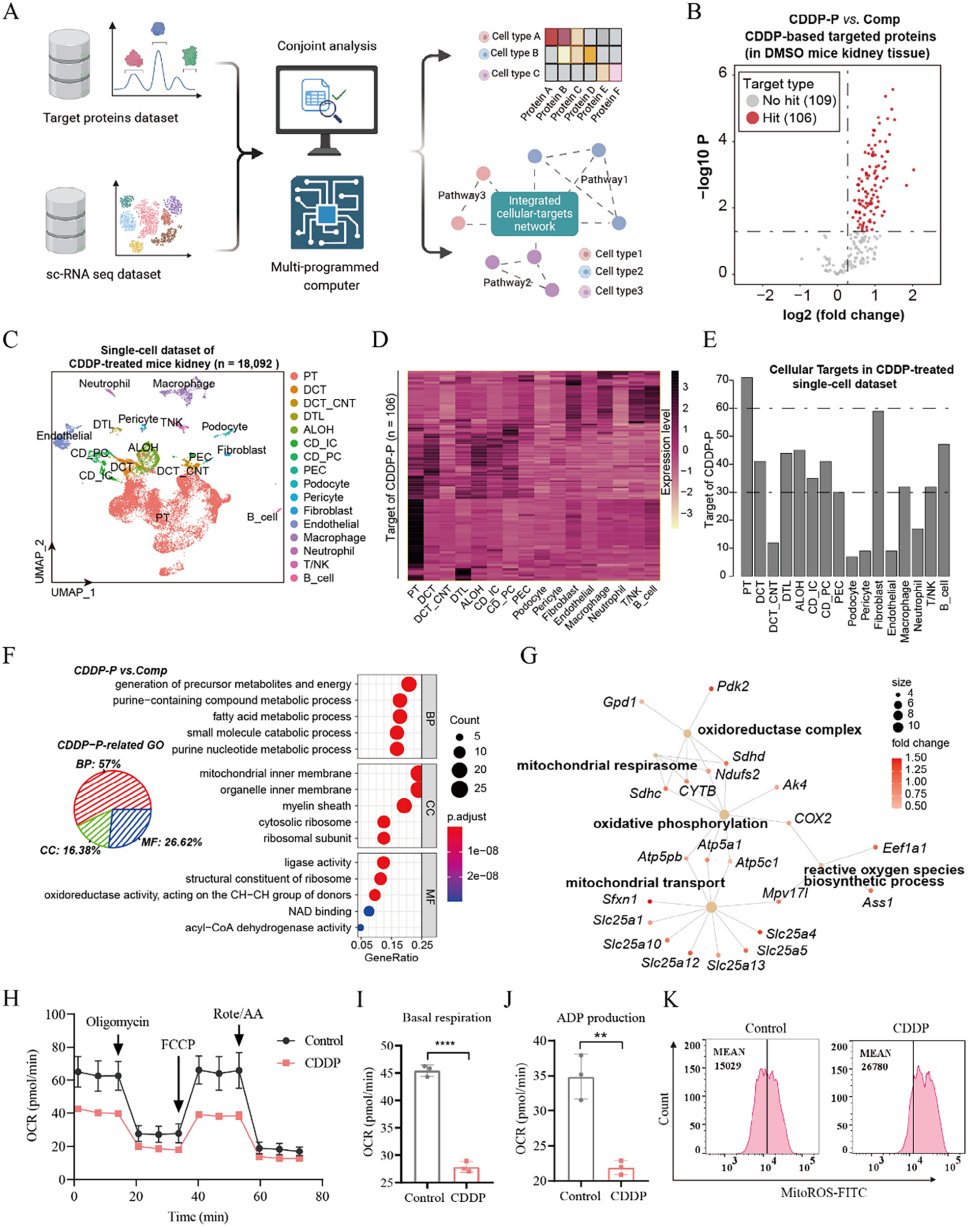
Cisplatin selectively activates biological pathways in PT cells. A) Schematic of STEP methodology profiling. B) Volcano plot illustrating the targeted proteins distribution captured by CDDP‐P (CDDP‐P vs. Comp). C) UMAP visualization demonstrating the distinct cellular types identified in the scRNA‐seq dataset of CDDP‐induced kidneys. D) The heatmap representing the relative expression levels of CDDP‐P‐targeted proteins across various cell types in the mouse kidney (CDDP‐P vs. Comp). E) The chart displaying the number of CDDP‐P‐targeted proteins detected in different cell types within the murine kidney (CDDP‐P vs. Comp). F) Gene Ontology analysis highlighting CDDP‐P‐targeted proteins associated with biological processes and functions. G) An illustration of the mitochondrial signaling pathways enriched by these targeted proteins (CDDP‐P vs. Comp). H) Oxygen consumption rate (OCR) is examined treated with vehicle (saline) or CDDP. I) Basal respiration capacity of OCR is examined in both groups (*n* = 3). J) ADP production capacity of OCR is examined in both groups (*n* = 3). K) MitoROS is detected by flow cytometry in both groups. The error bars indicate the means ± SD. The *p*‐values were determined by Student's *t‐*test; **p < 0.01, ****p < 0.0001 versus Control.

The research presented above identified that CDDP specifically binds to target proteins and elucidated its effects on cellular heterogeneity at the single‐cell level (Figures 2, 3). Fortunately, our previous research introduced STEP to facilitate a comprehensive assessment of targets at single‐cell resolution.^[^ ^19^ ^]^ Here, we integrated both the CDDP‐targeted proteins and scRNA‐seq datasets by STEP, facilitating the cellular targets profiling and the mediation of biological pathways influenced by CDDP (**Figure** **4**A). Initially, we screened differential proteins in chemical proteomic data (CDDP‐P vs. Comp group) and identified a total of 215 proteins, of which 106 may represent potential targets with *p* value < 0.05 and fold change > 1.2 (CDDP‐P vs. Comp) (Figure 4B). Subsequently, we integrated the potential targets identified in chemoproteomics with the scRNA‐seq dataset to evaluate the expression of cellular targets (Figure 4C,D). Furthermore, we delineated the distribution of protein targets at single‐cell resolution (Figure 4E). These findings indicate that PT cells exhibit the highest targets relative to other cell types, corroborating previous reports that PT cells serve as the primary target of CDDP in the kidney.^[^ ^34^ ^]^ Regarding the protein targets of CDDP in PT cells, gene ontology (GO) enrichment analysis revealed that CDDP had differential effects on various biological processes, primarily involving mitochondrial metabolic disturbances and respiration, oxidative stress, and ATP synthase, electron transfer activity (Figure 4F,G; Figure S4A,B, Supporting Information), which coincides well with the previous studies.^[^ ^6^, ^35^ ^]^ Importantly, we confirmed that CDDP inhibits mitochondrial respiration, ADP production, and electron transport activity through Seahorse experiments. These findings are consistent with our analytical results (Figure 4H–J; Figure S4C,D, Supporting Information). Furthermore, flow cytometry validated that CDDP induces ROS accumulation in mitochondria (Figure 4K; Figure S4E, Supporting Information). In addition, we found that CDDP target‐specific inhibitors significantly reduced ATP production in HK‐2 cells (Figure S4F,G, Supporting Information), thereby exhibiting similar inhibitory effects on mitochondrial energy metabolism. In summary, these findings indicate that CDDP selectively targets and impairs PT cells by interacting with mitochondria‐associated proteins, ultimately resulting in mitochondrial metabolic disturbances and damage, and further inducing nephrotoxicity.

### 2.5 Cisplatin‐Induced Proximal Tubule Cells Injury via Activating NF‐κB and Mitochondrial Stress Pathway

**Figure 5:**
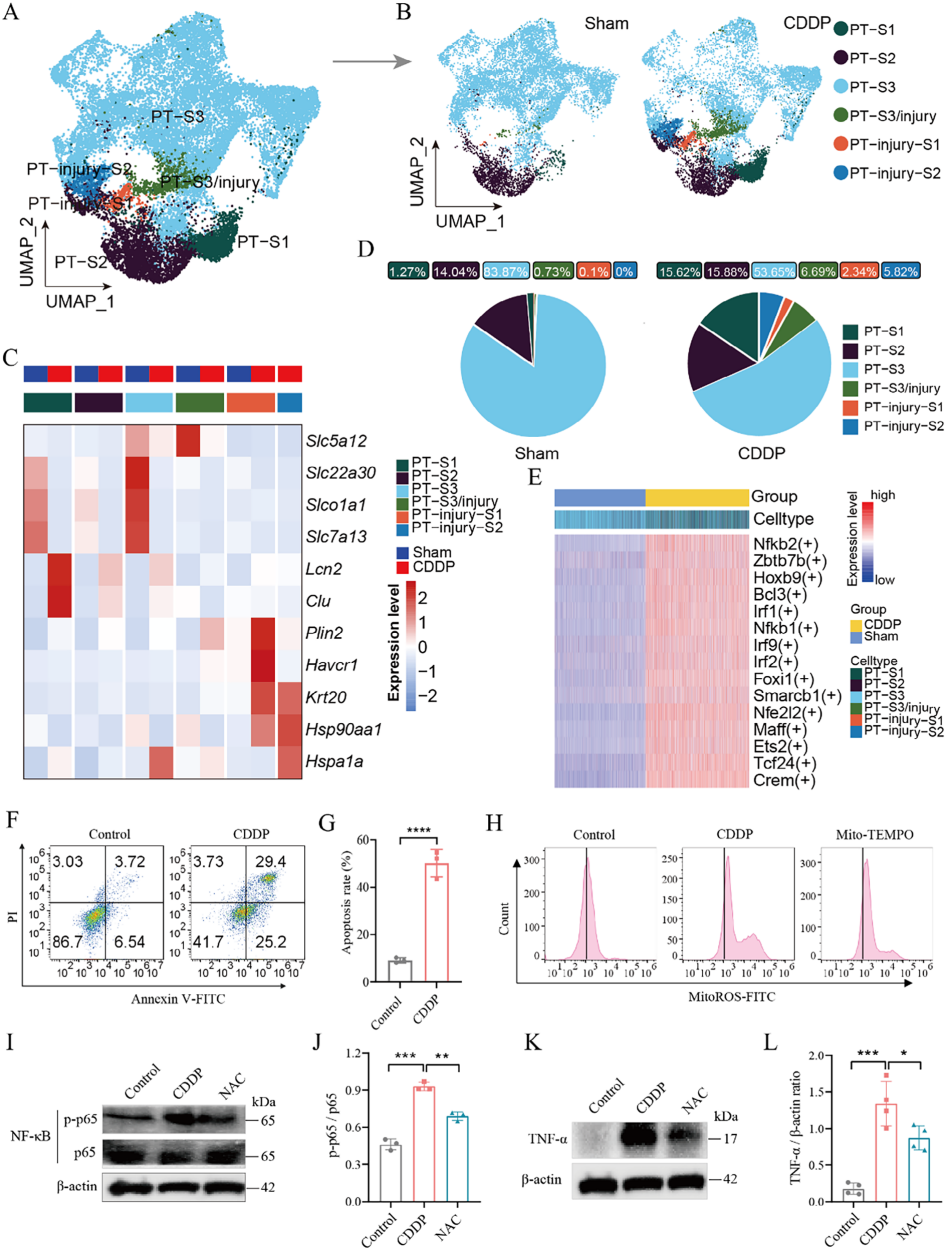
Cisplatin specifically induced proximal tubule (PT) cells injury via activating the NF‐κB pathway. A,B) UMAP reveals the various subtypes of PT cells in both the Sham and CDDP groups. C) The heatmap displays the expression level of the canonical marker genes among subtypes. D) The cellular composition of subtypes in the Sham and CDDP groups is illustrated. E) The analysis of transcription factor activity in the Sham and CDDP groups. F) Flow cytometry to verify the apoptosis of CDDP‐induced proximal tubular cells. G) Statistics for apoptotic cells corresponding to be Figure 5F (*n *= 3). The error bars indicate the means ± SD. The *p*‐values were determined by Student's *t*‐test; ****p < 0.0001 versus Control. H) Flow cytometry to MitoROS levels of HK‐2 cells incubated with vehicle (saline), CDDP, CDDP + Mito‐TEMPO (a mitochondrial‐targeted antioxidant). I) Expression of NF‐κB p‐p65/p65 in HK‐2 cells incubated with vehicle (saline), CDDP, CDDP + NAC groups. J) Statistics for CDDP‐induced NF‐κB p‐p65/p65 ratio corresponding to be Figure 5I (*n* = 3). K) Expression of TNF‐α in three groups. L) Statistics for CDDP‐induced TNF‐α expression corresponding to be Figure 5K (*n* = 4). The error bars indicate the means ± SD. The *p* values were determined by One‐way ANOVA; *p < 0.05, **p < 0.01, ***p < 0.001 versus CDDP group.

The proximal tubule, as the nephron segment most susceptible to injury, is characterized by abundant mitochondria and active transport capacity.^[^ ^36^ ^]^ Evidence demonstrated their tight functional coupling, in which dysfunction of transport activity impacts mitochondrial morphology.^[^ ^37^ ^]^ In this study, unsupervised clustering analysis of proximal tubule cells identified three healthy PT subpopulations (corresponding to S1–S3), and one mixed subcluster (S3/injury) comprising both S3 segment cells (*Slc5a2+*) and injury‐response cells (*Plin2+ Hspa1a+*) (**Figure** **5**A–C). In addition, two injured PT subclusters were annotated as injury‐S1(*Havcr1* ^+^) and injury‐S2 (*Krt20* ^+^) (Figure 5C). The PT cells in injured states showed downregulation of healthy PT markers including solute‐linked carriers (*Slc5a12* ^+^, *Slc7a13* ^+^), indicating cell differentiation. In addition, functional profiling revealed distinct signatures in these PT cells. PT‐injury‐S2 cells exhibited significant upregulation of response to ER, oxidative stress, and mitochondrial impairment‐relative pathways (eg., autophagy of mitochondrion). Moreover, PT‐injury‐S1 cells showed activity in toxin transport and protein export from the nucleus. Notably, both injury‐S1 and injury‐S2 subsets mediated cytochrome c release from mitochondria and apoptotic signaling (Figure S5A, Supporting Information). Cytochrome c can redistribute to the nucleus, cytosol, and extracellular compartment from the normally mitochondrial intermembrane space under stress‐induced conditions or specific pathological, where it regulated ROS dynamics and initiated apoptotic cell death.^[^ ^37^ ^]^ Furthermore, we observed a notably heterogeneous distribution of all PT cells between Sham and CDDP groups (Figure 5D). The PT‐S3 subcluster cells constituted the majority in both the Sham and the CDDP group. Our results also showed that injured PT subclusters were increased after CDDP treated (Figure 5D), and observed significant damage changes with higher injury scores in the CDDP‐treated group compared with Sham (Figure S5B,C, Supporting Information). To explore the transcription regulatory mechanism underlying renal injury, we further conducted transcription factor (TF) activity analysis and found increased activity of proinflammatory and apoptosis‐related TFs, including Nfκb1, Nfκb2, Zbtb7b, Hoxb9, and Bcl3 (Figure 5E). The expression changes in response to NF‐κB signal among the injury‐related PT cells were observed, as shown in Figure S5D (Supporting Information). Moreover, GSVA results for injury PTs (S1, S2) showed upregulation of pro‐inflammatory and ROS signaling (Figure S5E, Supporting Information). These findings suggest that CDDP may induce PT cell damage through NF‐κB activation and the ROS signaling pathway. In summary, we hypothesize that CDDP may cause oxidative stress by damaging mitochondria, activate NF‐κB to initiate an inflammatory response, and exacerbate renal toxicity.

To validate the aforementioned hypothesis, we conducted relevant molecular biochemical experiments. Initially, cellular studies revealed that CDDP induced apoptosis (Figure 5F,G). We observed that *N*‐acetylcysteine (NAC) could reverse intracellular ROS accumulation in HK‐2 cells induced by CDDP (Figure S5F,G, Supporting Information). Furthermore, we discovered that NAC can inhibit CDDP‐induced p‐p65 expression, thereby blocking transcriptional activation of NF‐κB (Figure 5I,J), subsequently reducing its downstream effector TNF‐α (Figure 5K,L). Moreover, the mitochondrial‐targeted antioxidant (Mito‐TEMPO) effectively scavenges CDDP‐triggered MitoROS, further validating the role of oxidative stress in this pathway (Figure 5H; Figure S5H, Supporting Information). The NF‐κB inhibitor (JSH23) potently suppressed CDDP‐induced nuclear translocation of NF‐κB in PT cells (Figure S5I, Supporting Information). These results indicate that CDDP may induce the accumulation of intracellular ROS, the phosphorylation of NF‐κB, and downstream inflammatory response.

### 2.6 Cisplatin Significantly Induces the Infiltration and Activation of Macrophages

**Figure 6:**
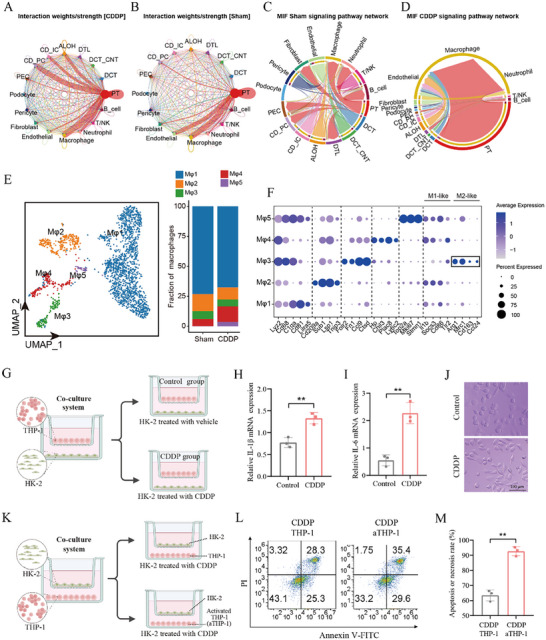
Cisplatin significantly induces renal infiltration and activation of macrophages. A,B) The chordal graph shows crosstalk between PT cells and other cells, colored basing on cell type and thickness degree in both Sham and CDDP groups. C,D) Communication probability of MIF signaling in cellular crosstalk between PT cells and other cells in both Sham and CDDP groups. E) UMAP reveals subtypes of macrophage (left) and cellular composition of subtypes (right). F) The dot plot shows the marker genes in the subtype of macrophage. G) Schematic diagram of the co‐culture system of THP‐1 and HK‐2 treated with vehicle (saline) and CDDP. H,I) QPCR assay to examine effects of CDDP‐injured HK‐2 on expression of inflammatory cytokines in THP‐1 of co‐culture system (*n* = 3). J) CDDP‐damaged HK‐cells medium supernatant activated THP‐1 (Scale bar = 100 µm). K) Schematic diagram of the co‐culture system of THP‐1 activated with phorbol 12‐myristate 13‐acetate (PMA, 50 ng mL^−1^) + IFN‐γ (20 ng mL^−1^)/LPS (100 ng mL^−1^), and HK‐2 treated with CDDP. L) Flow cytometry to examine the effects of activated THP‐1 on apoptosis of CDDP‐injured HK‐2. M) Statistics for apoptosis of CDDP‐injured HK‐2 corresponding to be Figure 6L (*n *= 3). The error bars indicate the means ± SD. The *p*‐values were determined by Student's *t*‐test; **p < 0.01 versus Control or CDDP THP‐1 group.

To elucidate cellular interaction changes between immune cells and PT cells from the Sham and CDDP groups, we utilized CellChat for crosstalk analysis. Compared to the Sham group, the weight/strength of cellular interactions among renal epithelial cells and macrophages in CDDP was elevated (**Figure** **6**A,B). We then compared the top 10 ligand–receptor (LR) pairs ranked by the relative contributions within their inferred signaling networks of the sham and CDDP group. Notably, several LRs associated with the macrophage migration inhibitory factor (MIF) pathway (*Mif‐Cd74/Cd44*, *Mif‐Cd74/Cxcr4*, and *Mif‐Ackr3*) showed decreased relative contribution rankings in CDDP, whereas those related to the SPP1 pathway (*Spp1‐Itgav/Itgb6*) displayed increased rankings (Figure S6A, Supporting Information). Moreover, *Cd74*, a MIF‐related gene, showed high expression in macrophages (Figure S6B, Supporting Information). We then specifically examined changes in the MIF communication network between the Sham and CDDP groups and found that CDDP increased MIF signaling in the crosstalk between PT and macrophage (Figure 6C,D). While MIF signaling is traditionally recognized for its role in inhibiting macrophage migration, it also facilitates the directed migration and recruitment of leukocytes.^[^ ^38^ ^]^ Therefore, we extended our analysis to investigate macrophages. Unsupervised clustering analysis of macrophage cells from the sham and CDDP groups identified five distinct subclusters (Mφ1‐Mφ5) (Figure 6E). Macrophage populations comprised four distinct subclusters expressing genes previously associated with resident macrophages (Mφ1: *Cd81^+^C1qa^+^*), regulated macrophages (Mφ2: *Cd68* ^+^ *Cd209a* ^+^) showed both pro‐inflammatory signatures and DC‐like signatures,^[^ ^39^ ^]^ and Mφ2‐like macrophages (Mφ3: *Fn1* ^+^ *Folr2* ^+^ *Mrc1* ^+^ *Cd163* ^+^), proliferating macrophages (Mφ5: *Mki67* ^+^ *Top2a* ^+^)), and infiltrating macrophages (Mφ4: *Plac8* ^+^ *Chil3* ^+^) (Figure 6F; Figure S6C, Supporting Information).^[^ ^40^ ^]^ Of these, infiltrating macrophages showed relatively higher expression of genes related to M1 polarization and pro‐inflammatory factors (*Il1b, Socs3, Cd86*, and *Tlr2*). In addition, our findings indicated an expanded distribution of infiltrating Mφ4 cells in the CDDP‐treated group compared to sham, suggesting that exposure to CDDP might facilitate the macrophage infiltration and polarization (Figure 6E,F; Figure S6D, Supporting Information). Consistent with that, GO pathways, such as macrophage migration and activation, were upregulated in Mφ4 (Figure S6E, Supporting Information). Moreover, Mφ3 subset (M2‐like macrophages) also downregulated pro‐inflammatory responses in CDDP (Figure S6F, Supporting Information).

To further investigate the impact of macrophages (THP‐1 cells) on PT cells (HK‐2 cells), we conducted a co‐culture system experiment. The co‐culture systems were divided into two groups: vehicle (saline) and CDDP groups (Figure 6G). Results indicated that the CDDP incubation group activated macrophages and upregulated expressions of IL‐1β and IL‐6 (Figure 6H,I). The co‐culture experiments demonstrated that macrophages were activated by CDDP‐damaged HK‐cells medium supernatant (Figure 6J). Additionally, the co‐culture systems were categorized into two groups: one group comprised resting macrophages (THP‐1) and HK‐2 cells treated with CDDP, while the other group consisted of activated macrophages (aTHP‐1 cells) and HK‐2 cells incubated with CDDP (Figure 6K). Results indicated that the activation of macrophages exacerbated CDDP‐induced toxicity to HK‐2 cells (Figure 6L,M). In summary, the results indicate that CDDP‐induced damage to PT cells may recruit and activate macrophages through the MIF signaling pathway. Subsequently, the infiltrating and activated macrophages further exacerbate the damage to PT cells caused by CDDP.

### 2.7 ICA Ameliorates CDDP‐Induced Nephrotoxicity While Synergistically Enhancing Anticancer Efficacy via Regulating Mitochondrial Metabolism and NF‐κB Pathway

**Figure 7:**
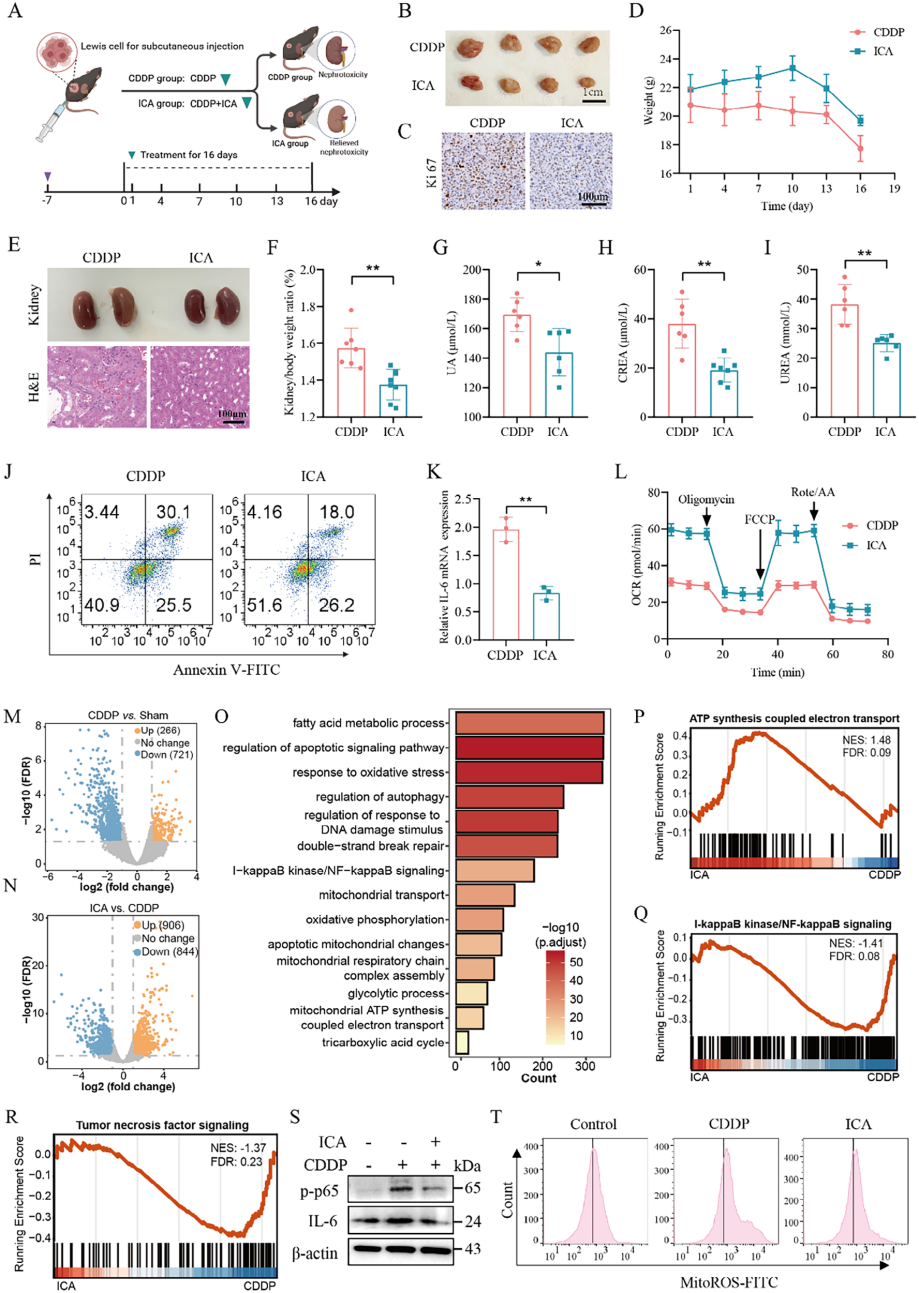
ICA ameliorates cisplatin‐induced nephrotoxicity while synergistically enhancing anticancer efficacy. A) Animal experimental protocol aimed at evaluating the effect of ICA in combination with CDDP on therapeutic efficacy and nephrotoxicity. B) Representative tumor morphology in alone CDDP or ICA in combination with CDDP, both the CDDP group and ICA group, after 16 days of treatment (scale bar = 1 cm). C) Immunohistochemical assay for Ki67 marker in both groups (scale bar = 100 µm). D) Statistics of body weight (*n* = 8–9). E) Representative kidney morphology, and H&E staining of renal tissues in both groups (scale bar = 100 µm). F) The kidney/body weight ratio (*n* = 7–9). G–I) Effect of ICA on CDDP‐induced UA, CREA, and UREA (*n* = 6). J) Flow cytometry to access the protective effects of ICA on the apoptosis or necrosis of CDDP‐induced HK‐2 cells. K) QPCR assay to examine effects of ICA on inflammatory cytokine IL‐6 in CDDP‐treated HK‐2 cells (*n* = 3). L) OCR is examined in ICA+CDDP or CDDP‐treated HK‐2 cells. M,N) Volcano plot of DEGs in CDDP versus Sham and ICA versus CDDP. O) BP enrichment of DEGs in ICA versus CDDP. P–R) Gene set analysis of several pathways. S) WB to detect the effect of NF‐κB and IL‐6 in Control, CDDP, CDDP + ICA treated HK‐2 cells (*n* = 3). T) Flow cytometry to MitoROS levels in Control, CDDP, CDDP + ICA treated HK‐2 cells (*n* = 3). The error bars indicate the means ± SD. The *p*‐values were determined by Student's *t*‐test; *p < 0.05, **p < 0.01 versus the CDDP group. NES, Normalized Enrichment Score; FDR, False Discovery Rate.

Currently, there is an urgent need to develop drugs that not only mitigate the toxicity associated with CDDP but also enhance its anticancer efficacy. ICA, an extract from the Chinese herbal medicine epimedium plant, exhibits significant antioxidant, anti‐inflammatory, and anticancer properties, particularly against HCC.^[^ ^26^, ^27^ ^]^ ICA is undergoing clinical trials in China for advanced HCC.^[^ ^41^ ^]^ Here, this study investigates whether ICA can alleviate CDDP‐induced renal toxicity and whether it may possess synergistic effects in the treatment of HCC. To investigate whether ICA ameliorates CDDP‐induced nephrotoxicity while synergistically enhancing anticancer efficacy, HCC tumor‐bearing mice were prepared to three groups: the model group, the CDDP group, and the CDDP combined with ICA treatment group (ICA group) (**Figure** **7**A). The model group data is not shown here.

Initially, the kidneys and tumors of HCC tumor‐bearing mice were dissected following the administration of CDDP (10 mg kg^−1^) or ICA (70 mg kg^−1^), and subsequent pathological assays were performed. The combination of CDDP and ICA treatment has been shown to effectively inhibit tumor growth compared to the CDDP group (Figure 7B; Figure S7A, Supporting Information) and reduce tumor cell proliferation (Figure 7C; Figure S7B, Supporting Information). Furthermore, this combination regimen reversed weight loss in mice, compared to the CDDP group (Figure 7D; Figure S7C, Supporting Information). The appearance and pathological staining of the kidney revealed significant improvements in the ICA group, compared to the CDDP group (Figure 7E). Additionally, our data indicate that ICA significantly improved the renal‐to‐body ratio (Figure 7F) and blood biochemical markers, specifically UA, CREA, and UREA (Figure 7G–I), suggesting that ICA significantly ameliorates CDDP‐induced nephrotoxicity. Simultaneously, it decreased in the liver‐to‐body ratio and levels of the biochemical markers aspartate aminotransferase (AST) and alanine aminotransferase (ALT) (Figure S7D–F, Supporting Information), indicating that ICA improves a certain degree of liver injury. In vitro studies demonstrated that ICA significantly alleviated CDDP‐induced apoptosis (Figure 7J; Figure S7G, Supporting Information). Furthermore, we observed that ICA inhibited the CDDP‐induced expressions of IL‐6 and IL‐1β (Figure 7K; Figure S7H, Supporting Information). Importantly, we confirmed that ICA mitigated the inhibitory effects of CDDP on mitochondrial respiration, ADP production, and electron transport activity, as evidenced by Seahorse experiments (Figure 7L; Figure S7I–L, Supporting Information). Therefore, ICA may ameliorate CDDP‐induced nephrotoxicity while synergistically enhancing anticancer efficacy in HCC tumor‐bearing mice.

To further investigate the underlying mechanisms by which ICA alleviates CDDP‐induced nephrotoxicity, we carried out RNA sequencing (RNA‐seq) on three groups: Sham, CDDP, and ICA groups. Correlation analysis revealed that intra‐group replicates exhibited higher correlation than inter‐group comparisons (Figure S7M,N, Supporting Information), indicating strong reproducibility of the sequencing data. Subsequently, differential expression analysis showed a multitude of differentially expressed genes (DEGs) in the ICA versus CDDP (1750 DEGs) and the CDDP versus Sham (987 DEGs). Specifically, the CDDP versus Sham identified 266 upregulated and 721 downregulated genes, whereas the ICA versus CDDP identified 906 upregulated and 844 downregulated genes (Figure 7M,N). The expression patterns of these DEGs showed high intra‐group consistency, further confirming the reliability of the data (Figure S7O,P, Supporting Information). To investigate the functional implications of these DEGs and the biological effects of ICA treatment, we performed functional enrichment analysis. DEGs from the CDDP versus Sham were enriched in essential pathways associated with inflammatory response, extracellular matrix organization, fatty acid metabolism, ATP metabolic process, fatty acid oxidation and glycolytic process (Figure S7Q, Supporting Information), By contrast, DEGs from the ICA versus CDDP were primarily enriched in fatty acid metabolic processes, regulation of apoptotic signaling pathways, and regulation of autophagy. Additionally, pathways related to NF‐κB signaling, mitochondrial transport and respiratory chain, oxidative phosphorylation were also significantly enriched (Figure 7O). These findings reveal that mitochondrial metabolism involves in CDDP‐induced nephrotoxicity and the therapeutic effects of ICA. Consistent with these results, our experimental data showed that CDDP inhibited mitochondrial basal respiration and ADP production, while ICA improved this damage (Figures 4, 7, 7L; Figure S7I–L, Supporting Information). Pathway analysis revealed that CDDP suppressed mitochondrial metabolism‐related processes, including oxidative phosphorylation, fatty acid β‐oxidation, the tricarboxylic acid (TCA) cycle, and the ATP transport chain, while concurrently activated inflammatory pathways such as NF‐κB signaling and Tumor necrosis factor signaling (Figure S7R–W, Supporting Information). Notably, ICA treatment effectively improved activity in these pathways (Figure 7P–R; Figure S8A, Supporting Information).

To further investigate whether ICA improves CDDP‐induced side effects by blocking the target proteins bound to CDDP, we performed CDDP‐P probe labeling‐related experiments for the binding domains of recombinant PC, MCCC1, PCCA, and SDHC proteins. Results showed no significant change in CDDP‐protein binding levels in the ICA‐treated group (Figure S8B, Supporting Information), indicating that ICA's protection maybe not mediated by blocking CDDP binding but rather by mitigating other downstream stress responses. Moreover, we found that ICA significantly attenuated CDDP‐triggered accumulation of MitoROS (Figure 7T; Figure S8C, Supporting Information), and ICA significantly rescued CDDP‐suppressed ATP generation (Figure S8D, Supporting Information). Simultaneously, ICA significantly inhibited CDDP‐induced phosphorylation of NF‐κB and downstream inflammatory response (Figure 7Q–S; Figures S8E,F,S9A, Supporting Information). Taken together, ICA fails to directly block CDDP‐binding proteins; rather, it mitigates CDDP‐induced nephrotoxicity through the improvement of CDDP‐elicited inflammatory responses and mitochondrial dysfunction, and simultaneously exerts a synergistic effect to boost cisplatin's anticancer efficacy.

### 2.8 Integrated Transcriptomics and Metabolomics Reveal That ICA Alleviates the Inflammatory Response and Mitochondrial Disturbances Induced by CDDP

**Figure 8:**
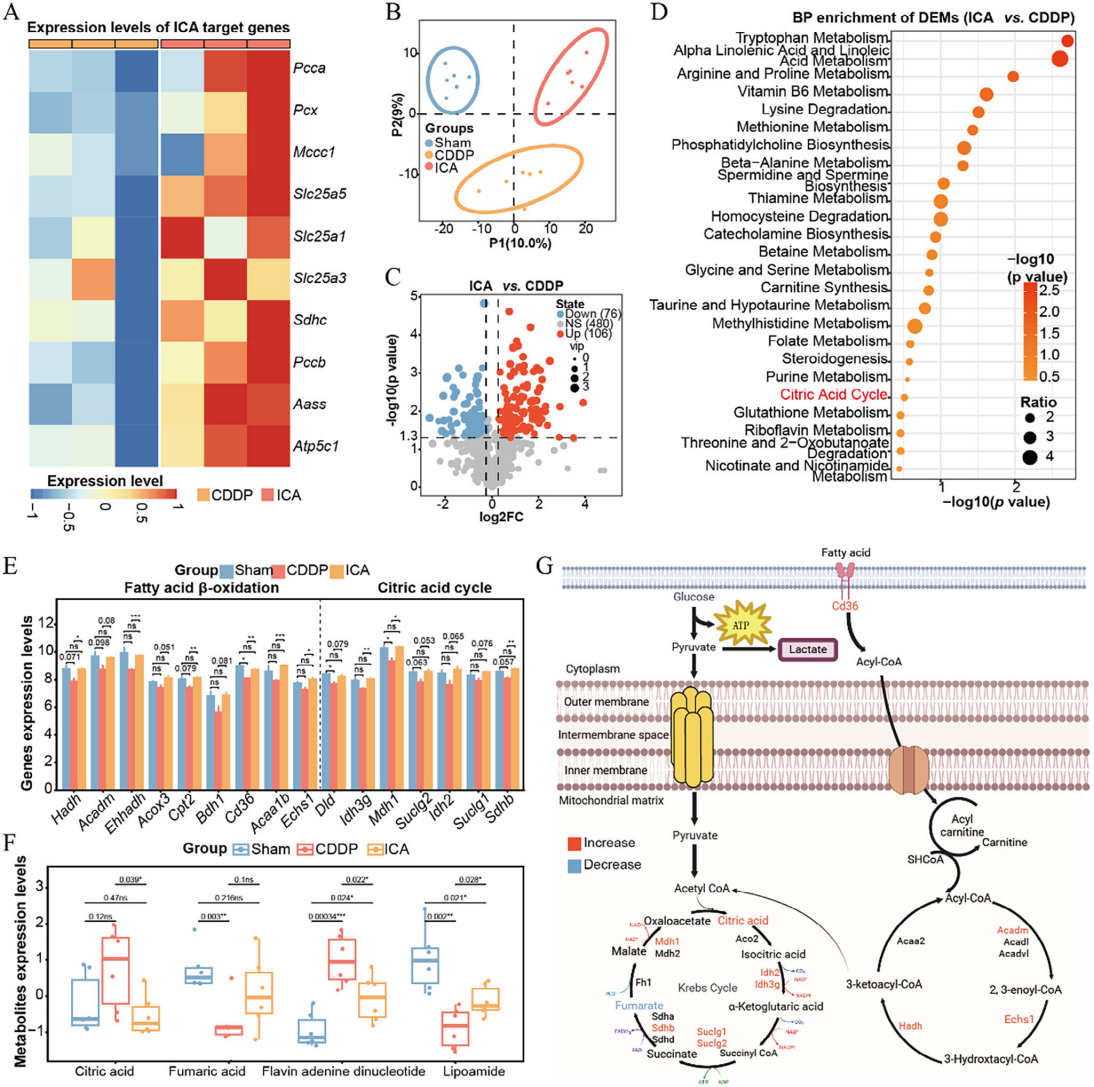
ICA alleviates CDDP‐induced inflammatory response and mitochondrial disturbances. A) Expression level of CDDP‐targeted genes in the CDDP and ICA group. B) Sample distribution of OPLS‐DA analysis. C) Volcano plot shows the distribution of DEMs in ICA versus CDDP. D) KEGG enrichment of DEMs in ICA versus CDDP. E) Expression distribution of genes related to metabolic pathways. F) Contents of metabolites related to the citric acid cycle. G) Schematic diagram of genes and metabolites in the process of this study (red represents upregulation and blue represents downregulation) (RNA seq *n *= 3 per group; metabolites *n *= 6 per group). The *p*‐values were calculated by Student's t‐test; ns: not significant, *p < 0.05, **p < 0.01, *** *p* < 0.001.

Our experimental findings and RNA‐seq analysis revealed that CDDP inhibits mitochondrial‐related metabolic processes in the kidney, while ICA remodels the mitochondrial metabolic microenvironment. Notably, CDDP primarily targets the citrate cycle and oxidative phosphorylation, whereas ICA ameliorates these impairments by modulating the associated pathways (Figures 2, 7P; Figure S8A, Supporting Information). Specifically, ICA upregulated expressions of key target genes involved in some metabolic processes (**Figure** **8**A). To validate these findings, we performed metabolomic analysis to examine changes in metabolism‐related pathways. Quality control (QC) samples in both positive and negative modes exhibited high correlations (Figure S9B,C, Supporting Information), and principal component analysis (PCA) clearly separated the QC samples, Sham group, CDDP group, and ICA group (Figure S9D,E, Supporting Information), indicating the reliability and stability of the data. Orthogonal partial least squares‐discriminant analysis (Ortho PLS‐DA) further distinguished the Sham, CDDP, and ICA treatment groups (Figure 8B). We then conducted differential metabolite analysis by merging metabolites identified in positive and negative modes. CDDP treatment led to significant alterations in 346 metabolites (|log2 fold change| > 0.26, *p* < 0.05) (Figure S9F, Supporting Information), while ICA treatment of CDDP‐induced nephrotoxicity resulted in significant changes in 182 metabolites (Figure 8C). Differential metabolite expression patterns showed high consistency within groups with the ICA‐treated group displaying an overall metabolic profile similar to the Sham group, suggesting that ICA effectively mitigates CDDP‐induced metabolic disorders (Figure S9G, Supporting Information). To investigate the biological pathways involved, we carried out KEGG enrichment analysis on metabolites from CDDP versus Sham and ICA versus CDDP comparisons. CDDP versus Sham metabolites were enriched in pathways, including arginine and proline metabolisms, aspartate metabolisms, and the urea cycle, along with glutathione metabolisms and branched‐chain fatty acid oxidation (Figure S9H, Supporting Information). Metabolites in the ICA versus CDDP comparison were also enriched in glutathione metabolism, in addition to the citric acid cycle (Figure 8D). Based on these results and the above findings, we focused on the fatty acid β‐oxidation and citric acid cycle pathways to evaluate changes in the expression of related genes that were suppressed in the CDDP treatment group but reactivated by ICA treatment (Figure 8E). Differential metabolites within the citric acid cycle exhibited varied changes: CDDP increased the accumulation of citric acid and flavin adenine dinucleotide while reducing fumaric acid and lipoamide levels. ICA treatment effectively corrected these metabolic imbalances (Figure 8F). To provide a comprehensive view, we mapped the altered metabolites and genes onto classical pathways of the TCA cycle and fatty acid β‐oxidation (Figure 8G). Collectively, results demonstrate that ICA targets mitochondrial metabolism‐related genes, activates their expression, and restores CDDP‐induced metabolic disorders, particularly in fatty acid β‐oxidation and citric acid cycle pathways.

## 3 Discussion

CDDP is extensively utilized in the treatment of various cancer types; however, its application can be compromised by severe adverse effects in healthy tissues, particularly nephrotoxicity, which affects approximately one‐third of treated patients.^[^ ^25^, ^42^, ^43^, ^44^ ^]^ Identifying CDDP‐binding targets to mitigate these toxicities without reducing tumor control remains a significant clinical challenge. Additionally, an optimal therapeutic approach should ideally synergize with CDDP to promote tumor regression while safeguarding renal and other physiological functions. In this study, we identified that CDDP directly binds to target proteins, leading to nephrotoxicity, and further integrated chemoproteomics and scRNA‐seq datasets to elucidate the underlying toxic mechanisms. Furthermore, we provided evidence that the administration of ICA effectively prevented CDDP‐induced nephrotoxicity in tumor‐bearing mice. These beneficial effects were noted alongside a synergistic enhancement of the anti‐cancer properties of CDDP.

Chemoproteomics demonstrated that CDDP directly binds to mitochondrial proteins, including PCCA, PC, ATP5F1C, SLC25A5, and SDHC. Subsequently, the STEP‐based analytical method^[^ ^19^ ^]^ demonstrates that CDDP selectively interacts with mitochondria‐associated proteins in PT cells, ultimately resulting in mitochondrial respiration and metabolism, intracellular ROS accumulation, inflammatory responses, and apoptosis. These results are consistent with previous single‐nucleus RNA sequencing studies on CDDP‐induced nephrotoxicity, which revealed transcriptional alterations in potential PT injury mechanisms, including inflammation and lipid metabolism pathways.^[^ ^45^ ^]^ Other studies have demonstrated that the cytotoxic mechanisms of CDDP involve ROS‐mediated oxidative stress, mitochondrial disorder, and DNA adduct formation, which ultimately lead to apoptosis.^[^ ^34^, ^46^, ^47^, ^48^ ^]^ In contrast to previous studies that investigated the mechanisms of CDDP‐induced nephrotoxicity through proteomics, bulk transcriptomics, and metabolic analyses,^[^ ^49^, ^50^ ^]^ our findings provide a comprehensive elucidation of the targeting mechanisms by which CDDP induces injury in PT cells via multiple pathways, ultimately leading to severe renal toxicity.

In the PT cells of the CDDP group, we identified the activation of translation factors such as NFκB2, NFκB1, IRF1, and FOXI1, along with the upregulation of pathways including NF‐κB and IL‐6/JAK/STAT3 signaling. The injured PT cells induced an inflammatory response via activating the NF‐κB signaling pathway.^[^ ^51^ ^]^ These alterations may promote the synthesis and release of inflammatory factors, thereby leading to renal toxicity.^[^ ^52^ ^]^ Moreover, a positive feedback loop involving inflammatory factor secretion from renal PT cells, NF‐κB activation, and mitochondrial dysfunction may further exacerbate renal damage following CDDP treatment.^[^ ^53^, ^54^, ^55^ ^]^ Collectively, our findings suggest that CDDP may induce mitochondrial dysfunction by triggering NF‐κB related with inflammatory response, ultimately contributing to PT cell damage.

To address the significant clinical challenge of detoxifying CDDP‐induced nephrotoxicity without compromising its anticancer efficacy, we have identified a number of potential candidates from traditional Chinese medicine and natural products that may mitigate CDDP‐induced nephrotoxicity.^[^ ^56^, ^57^ ^]^ We found that a natural antitumor agent, ICA gel, has been utilized as a clinical treatment for advanced HCC in China, demonstrating favorable safety and effectiveness in clinical application.^[^ ^26^, ^41^, ^58^ ^]^ Notably, the administration of ICA may mitigate CDDP‐induced damage to PT cells both in vivo and in vitro. ICA has been shown to enhance mitochondrial respiration and decrease the accumulation of intracellular ROS. Mitochondrial respiration serves as a pivotal energy source for ATP production in PT cells.^[^ ^59^, ^60^, ^61^ ^]^ Recent studies indicate that impaired mitochondrial respiration leads to redox imbalances and inflammatory responses, which are critical in the pathogenesis of nephrotoxicity.^[^ ^62^, ^63^ ^]^ CDDP promotes ROS accumulation within the mitochondria of PT cells, disrupts mitochondrial electron transport, diminishes antioxidant capacities, and ultimately results in ROS‐mediated injury and cell death in PT cells.^[^ ^48^, ^64^ ^]^ The analysis of ligand–receptor pairings revealed crucial intercellular communication between macrophages and PT cells,^[^ ^21^ ^]^ highlighting the role of this interaction in damage‐induced inflammation by CDDP, suggesting a potential pathway for the recruitment and activation of macrophages. Collectively, our findings demonstrate that ICA effectively reverses the mitochondrial respiratory imbalance, intracellular ROS accumulation, and macrophage infiltration induced by CDDP. Moreover, ICA appears to collaborate with CDDP to exert anti‐cancer effects.

Moreover, we integrated transcriptomic and metabolomic analyses to further elucidate the protective mechanisms of ICA in kidneys by CDDP‐induced damage. Transcriptomic results indicated that ICA significantly inhibited CDDP‐induced mitochondrial damage and NF‐kB activation. Previous studies investigating the detoxification of CDDP nephrotoxicity have reported analogous findings.^[^ ^6^, ^65^ ^]^ Concurrently, metabolic analyses revealed that ICA mitigates mitochondrial metabolic disturbances induced by CDDP, particularly those associated with the tricarboxylic acid cycle, citrate cycle, and oxidative phosphorylation.^[^ ^66^, ^67^ ^]^

This study has several limitations. First, though we employed a 10 mg kg^−1^ CDDP regimen based on preliminary dose‐ranging tests, this dosage lies beyond the generally accepted therapeutic window. In future studies, we plan to explore the use of a lower‐dose, prolonged‐duration regimen or a clinically relevant dosage to further improve the model's robustness and interstudy comparability. Second, while this study identified the direct binding targets of CDDP, further research is needed to elucidate the binding mechanisms and mitochondrial‐related biological functions of these target proteins. Furthermore, given that CDDP triggers mitochondrial damage through multi‐target and multi‐pathway mechanisms, subsequent research should utilize multiple synergistic validation methods to examine how CDDP‐mediated targets impact mitochondrial function. Additionally, the mechanisms by which ICA mitigates CDDP‐induced nephrotoxicity require further in‐depth investigation. Finally, additional studies are warranted to uncover the mechanisms by which ICA synergistically enhances the chemotherapeutic efficacy of CDDP.

## 4 Conclusion

In summary, our study demonstrated that ICA can mitigate CDDP‐induced renal toxicity while enhancing its antitumor efficacy. Given the established safety and efficacy of ICA in China, our findings suggest its potential clinical application for patients with cancer undergoing CDDP treatment. Furthermore, we identified that CDDP‐bound molecular targets induced mitochondrial dysfunction, thereby inducing renal toxicity, offering valuable insights into the mechanisms underlying CDDP‐induced renal toxicity. This also highlights the importance of potential detoxification agents for clinical screening or development aimed at alleviating the global burden of chemotherapy.

## 5 Experimental Section

### Materials and Reagents

CDDP and ICA were obtained from Sigma (St. Louis, USA). *N‐*acetylcysteine (NAC) was acquired from AbMole BioScience (USA). The reagents for the click chemistry reactions, including TAMRA‐azide, THPTA, and Biotin‐azide, were sourced from Click Chemistry Tools (USA). NaVc and CuSO_4_ were obtained from Sigma Aldrich (St. Louis, USA). Mito‐TEMPO and JSH23 were obtained from MedChemexpress (USA). Some assay kits for AST, ALT, UA, CREA, and UREA were sourced from Beijian Xinchuang Yuan Technology (Beijing, China). The recombinant PC (Cat# PDB0897), PCCA (Cat# PDB0602), MCCC1 (Cat# PT13556), and SDHC (Cat# PT8057) protein fragments were sourced from Shanghai Qiming Biotechnology Co., LTD. Mitochondrial stress assay kits were acquired from Agilent (California, USA). Primary antibodies: NF‐kB p65 (Cat# 10745‐1‐AP), NF‐kB p‐p65 (Cat# 82335‐1‐RR), TNF‐α (Cat# 17590‐1‐AP), IL‐6 (Cat# 21865‐1‐AP), PCCA (Cat# 21988‐1‐AP), PC (Cat# 16588‐1‐AP), MCCC1 (Cat# 14861‐1‐AP) SLC25A5 (Cat# 17796‐1‐AP), ATP5F1C (Cat# 10910‐1‐AP), Histone H3 (Cat#17168‐1‐AP) and β‐actin (Cat# 66009–1‐Ig) were sourced from Proteintech (Chicago, USA). Atpenin A5 and Benzophenone were obtained from Santa Cruz Biotechnology.

### Cell Culture or Co‐Culture System and Viability Assays

Hepa1‐6, THP‐1, and HK‐2 cells, obtained from the Chinese National Cell Bank (Beijing, China), were cultured in Dulbecco's Modified Eagle Medium supplemented with 10% fetal bovine serum and maintained under appropriate atmospheric conditions. Cell viability was determined by using the Cell Counting Kit‐8 (CCK‐8) (Dojindo, Japan). For the co‐culture system, THP‐1 and HK‐2 cells were co‐cultured in a transwell chamber.

### Experimental Models of Tumor‐Bearing Mice

To validate the reliability of the model established by administering CDDP, preliminary experiments were conducted to optimize the treatment protocol. All animal experimental procedures were approved by the Institutional Animal Care and Use Committee of this institution. (Ethics Approval Number: AUP‐230714‐WJG‐619‐01). Animal procedures were conducted using male C57Bl6/J mice (8 weeks), obtained from Vital River (Guangzhou, China). The mice were provided a standard laboratory diet, maintained under consistent environmental conditions. Tumor‐bearing mice were prepared by Hepa1‐6 cells. After 7 days, when tumors reached ≈50–100 mm^3^, the mice with tumors were assigned to three groups. Mice were administered either normal saline (Sham group), cisplatin (CDDP, 10 mg kg^−1^, intraperitoneal (i.p.)) (CDDP group), or a combination of CDDP (10 mg kg^−1^ i.p. once weekly) and ICA (70 mg kg^−1^, intragastrical, once daily) (ICA group) for a total of 16 days. Following this treatment period, mice were euthanized, kidney and tumor tissues were collected. Tissues were fixed in 4% formaldehyde for H&E staining; the other part was frozen in a −80 °C freezer. Tumor growth was assessed through immunohistochemical staining assays. Images were obtained using a brightfield light microscope.

### Serum Biochemical Analysis

AST, ALT, UA, UREA, and CREA were measured using commercial kits in conjunction with an automatic biochemistry analyzer (TBA‐120FR, Toshiba, Japan).

### Fluorescence Labeling Experiments In Vitro

For fluorescence labeling experiments,^[^ ^18^, ^68^, ^69^ ^]^ kidney lysate was prepared and incubated with CDDP‐P (12.5–100 µmol L^−1^) or vehicle (saline) for 3 h. Subsequently, the samples were treated with a click chemistry mixture (NaVc, THPTA, CuSO_4_, and TAMRA‐azide). Following this, proteins were precipitated using pre‐chilled acetone and re‐dissolved in loading buffer by an SDS‐PAGE gel for separation, and visualization was performed using fluorescence scanning with a laser scanner. The gel was stained by using Coomassie Brilliant Blue (CBB).

For competitive fluorescence labeling experiments, kidney lysate was pre‐incubated with CDDP or vehicle, followed by treatment with CDDP‐P. The remaining procedures were consistent with the above experiments.

For recombinant protein fluorescence labeling, proteins were incubated with CDDP‐P, followed by a click reaction using a click cocktail. Samples were separated by SDS‐PAGE and visualized using the same procedure described above. In competitive experiments, samples were pre‐incubated with ICA prior to probe addition, followed by the same processing steps as outlined.

### Pull‐Down Targets Identification by LC/MS/MS and Data Analysis

For the target profile by LC/MS/MS,^[^ ^31^, ^70^ ^]^ kidney lysate was treated with CDDP or vehicle, followed by exposure to CDDP‐P for an additional 2 h. The sample was subjected to click reaction for 2 h at RT. The sample was precipitated, resolubilized in phosphate‐buffered saline (PBS) containing SDS, and treated with streptavidin beads. Beads were gently washed with a series of buffers. The protein‐bound beads were reduced, alkylated, and digested with trypsin. The sample was labeled with tandem mass tag (TMT) reagents (Thermo Scientific, USA), and subsequently analyzed using LC/MS/MS (Thermo Scientific, USA).

Based on the TMT signals from the CDDP‐P group and the CDDP‐P + CDDP (Comp) group, *p*‐values (CDDP‐P/Comp) were calculated using a Student's *t‐*test, following previously established methodologies. Target proteins were selected according to an absolute fold change greater than 1.2 and *p*‐values less than 0.05. GO enrichment analysis was conducted on the selected proteins using the “clusterProfiler” package in R.^[^ ^71^ ^]^

### Single‐Cell Suspensions and scRNA‑Seq

Fresh kidney tissues were harvested and subsequently cut into small fragments. These kidney fragments were digested. After digestion, the samples were filtered, centrifuged, and resuspended. Red blood cells were eliminated to achieve single‐cell suspensions. According to the official protocol from 10× Genomics, single‐cell suspensions were used to prepare sequencing libraries with the Single Cell 3′ Reagent Kit v3.1 (10× Genomics). The prepared libraries were then sequenced on the Illumina NovaSeq 6000 platform (Illumina, San Diego, CA, USA).

### scRNA‐Seq Dataset Analysis

DEGs for each cell type were identified using the Seurat package with default parameters. Genes that met the criteria of |log2(FC)| ≥ 0.25 and an adjusted *p‐*value < 0.05 were classified as DEGs. Functional enrichment analysis of the DEGs was conducted employing the clusterProfiler package (version 4.0.0), with multiple hypothesis testing adjusted via the Benjamini–Hochberg method.

### Cellular Protein Targets Analysis

The AverageExpression function was employed in Seurat to generate an averaged feature expression matrix for chemoproteomics‐identified targets across all cell types in the scRNA‐seq datasets. Subsequently, the averaged feature expression matrix was raw‐scaled to enable cross‐cell type comparison of mean feature expression values. For each target, if a cell type exhibited the highest scaled value relative to other types, that protein target was designated as cell‐type‐associated. After iterating through all candidate targets, acquired the distribution profiles were acquired within the cellular context.

### Biological Function Profiling

GO analysis was conducted by the “clusterProfiler”R package (version 3.18.1), tailored to the profiles of cellular targets. *p*‐values were calculated via the hypergeometric test and adjusted for multiple comparisons with the Benjamini–Hochberg (BH) procedure. The BP (Biological Process) category was selected to characterize functional profiles and visualized using the count of enriched proteins and the adjusted *p*‐value. The compareCluster function was employed to create comparison gene clusters for functional profiles within the scRNA‐seq dataset, while the cnetplot function was utilized to visualize concordant and divergent functional pathways alongside the relevant protein targets. Furthermore, gene set enrichment analysis (GSEA) was performed using the entire DEGs profile from the comparison of the CDDP and sham groups in bulk RNA‐seq. Additionally, the enrichment scores of these pathways were assessed using the DEGs profile from the CDDP versus sham groups within kidney cells.

### Cell–Cell Intercellular Networks and Gene Regulatory Network Analysis

The cellular crosstalk was systematically analyzed through ligand–receptor interactions utilizing the CellChat package. Specifically, the functions netVisual_aggregate and netVisual_chord_gene were employed to evaluate the crosstalk between PT cells and immune cells. An analysis of gene regulatory networks in hepatocytes was conducted using the PySCENIC package. The regulatory activities of transcription factors were visualized using the pheatmap function.

### Seahorse Assay

OCR assay was measured with a Mito Stress Test kit in vehicle or CDDP‐treated HK‐2 cells. Subsequent experiments adhered to the manufacturer's protocols. Data processing was analyzed using the Seahorse XFe Wave software.

### Flow Cytometry to Measure ROS, MitoROS, or Apoptosis

For the measurement of ROS and MitoROS, HK‐2 cells treated with vehicle, CDDP, or a combination of CDDP and ICA or NAC or Mtio‐TEMPO were gently washed and subsequently incubated in a binding buffer containing the ROS or MitoROS dye. Following this incubation, the samples were analyzed using flow cytometry.

For the cell apoptosis assay, HK‐2 cells treated with vehicle, CDDP, or a combination of CDDP and ICA or NAC and incubated in a binding buffer containing the Annexin V and propidium iodide dye. Following this incubation, the samples were analyzed using flow cytometry within 1 h.

### Western Blotting Analysis

Soluble protein was obtained from kidney tissues or HK‐2 cells, followed by loading onto an SDS‐PAGE gel for electrophoretic separation and subsequent transfer to polyvinylidene fluoride membranes. Samples were then incubated with primary antibodies and secondary antibodies. Finally, the protein signal was visualized by enzyme‐linked chemiluminescence and analyzed using ImageJ software.

For the CETSA‐WB, soluble protein lysates pre‐treated with CDDP were aliquoted into tubes and then heated at the specified temperatures. Following centrifugation, the soluble supernatants were subjected to Western blot analysis.

### Quantitative Real‐Time PCR (QPCR) Analysis

Total RNA was extracted from samples, and subsequently reverse‐transcribed into complementary DNA. QPCR was performed employing synthetic primers and SYBR Green Master Mix (YEASEN, China). The primer sequences for IL‐1β and IL‐6 are detailed in Table S1 (Supporting Information).

### Bulk RNA‐Seq Analysis

Total RNA was extracted from kidney samples (Sham, CDDP, and ICA groups), and poly(A)‐tailed transcripts were enriched using the poly(A) selection method. The enriched RNA was reverse‐transcribed into cDNA, and sequencing libraries were prepared prior to RNA sequencing on the Illumina NovaSeq 6000 platform with PE150 (Pair end 150 bp). Raw sequencing data were processed using fastp to filter low‐quality reads, retaining only high‐quality reads for subsequent analysis. The filtered reads were then aligned to the reference genome using HISAT2 (v2.1.0). Transcript quantification for each sample was performed with FeatureCounts, and the resulting data were normalized and analyzed for differential expression using the R package edgeR. Genes meeting the criteria of |log2 fold change| > 1 and *p*‐value < 0.05 (adjusted for multiple testing) were identified as DEGs. Functional enrichment analysis of DEGs was performed using the R package clusterProfiler to identify enriched biological pathways and processes.

### Metabolomic Analysis

Kidney sample preparation and metabolite extraction were conducted basing on the previously protocols. Metabolites were analyzed by using LC/MS/MS. Raw metabolite data were processed by using Compound Discoverer 3.1, and relative metabolite quantification was based on peak area measurements. Sample data were normalized using the R package MetNormalizer. PCA and orthogonal partial least squares‐discriminant analysis (OPLS‐DA) were conducted using the R package ropls (v1.30.0). Group means were compared using a *t‐*test, and multiple hypothesis testing was adjusted using the p.adjust function. Enrichment analysis of differential metabolites was performed with MetaboAnalyst 6.0.

### Molecular Docking Model

Protein crystals were sourced from the Protein Data Bank (PDB). For the docking process, proteins were first subjected to dehydration and hydrogenation using Discovery Studio Client. Molecular docking was performed with Pyrx‐0.8 software, followed by visualization and mapping using PyMOL.

### Statistical Analysis

Raw data were assessed for completeness and quality first. For scRNA‐seq data, log‐normalization was applied. Outliers were removed based on the distribution of feature RNA numbers and total RNA counts per cell. For bulk RNA‐seq data, normalization was conducted using the trimmed mean of *M*‐values method. Probabilistic quotient normalization was applied to normalize the metabolomics data using the implementation available in the MetaX. Data were presented as means + standard deviation (SD). Wilcoxon rank‐sum tests, exact test, and unpaired two‐tailed Students *t‐*tests were used to access statistical significance in both groups. *p*‐value was adjusted for multiple comparisons by using the false discovery rate (FDR) or the Bonferroni correction method. Statistical analyses were performed using GraphPad Prism 8.0 and R (v4.2.0), while schematic diagrams were generated using BioRender.com.

## Conflict of Interest

The authors declare no conflict of interest.

## Author Contributions

P.L., J.C., Y.A., and K.M. contributed equally to this work. Y.G., J.W., Q.Z., and P.L. designed and conceived the project. P.L., J.C., K.M., and Y.A. conducted the animal experiments, collected the results, and wrote the original draft. W.Z., W.L., J.L., and W.Z. conducted the animal experiments. H.H. and W.H. carried out the cell and molecular experiments. J.H., T.C., and S.F. performed the bioinformatics analysis. S.Y., M.H., H.H., and J.L. contributed to software and formal analysis, while J.W., P.L., J.C., and Q.Z. revised manuscript. All authors contributed to the discussion and interpretation of the results. All authors reviewed and approved the final version of the manuscript.

## Supporting information



Supporting Information

## Data Availability

The data that support the findings of this study are available from the corresponding author upon reasonable request.
