# Supplementary material for: Icaritin Ameliorates Cisplatin‐Induced Mitochondrial Metabolic Dysfunction‐Associated Nephrotoxicity and Synergistically Potentiates Its Antitumor Efficacy
Source: Adv Sci (Weinh). 2025 Nov 30;13(7):e06712. doi: 10.1002/advs.202506712 (PMC12866805; doi:10.1002/advs.202506712)
Supplement: Supplementary file 1 — Supporting Information [file ADVS-13-e06712-s001.docx]

Supporting Information

Icaritin Ameliorates Cisplatin-Induced Mitochondrial Metabolic Dysfunction-Associated Nephrotoxicity and Synergistically Potentiates Its Antitumor Efficacy

***Authors:*** *Piao Luo^1 ,2, #,^ * , Junhui Chen^4, #^, Yehai An^1, #^, Kun Meng^3, #^, Wei Zhou^2^, Wenhui Li^1^, Jing Liu^3^, Wentong Zhao^3^, Weiyi He^1, 3^, Ting Cao^1^, Jingnan Huang^3^, Sha Feng^1^, Shiguang Yang^1^, Hongling Hu^1^, Jiaxian Liao^1^, Hengkai He^4^, Mingjing Hao ^4^, Qian Zhang^1,^ *, Jigang Wang^1, 3, 4,^ *, Yue Gao^2,^ **

***Affiliations:***

1. Guangdong Basic Research center of Excellence for Integrated Traditional and Western Medicine for Qingzhi Diseases, Guangdong provincial Key Laboratory of Chinese Medicine pharmaceutics, School of Traditional Chinese Medicine and School of pharmaceutical Sciences, Southern Medical University, Guangzhou 510515, Guangdong, China.

2. Department of Pharmaceutical Sciences, Beijing Institute of Radiation Medicine, Beijing 100850, China

3. State Key Laboratory for Quality Ensurance and Sustainable Use of Dao-di Herbs, Artemisinin Research Center, and Institute of Chinese Materia Medica, China Academy of Chinese Medical Sciences, Beijing 100700, China.

4. Department of Pulmonary and Critical Care Medicine, Shenzhen Institute of Respiratory Diseases, and Shenzhen Clinical Research Centre for Geriatrics, Shenzhen People's Hospital; First Affiliated Hospital of Southern University of Science and Technology; Second Clinical Medical College of Jinan University, Shenzhen 518020, Guangdong, China.

#These authors made equal contributions to this work.

*Corresponding authors: luopiao@smu.edu.cn (Piao Luo); zhangqian408@yeah.net (Qian Zhang); jgwang@icmm.ac.cn (Jigang Wang); gaoyue@bmi.ac.cn (Yue Gao)

**
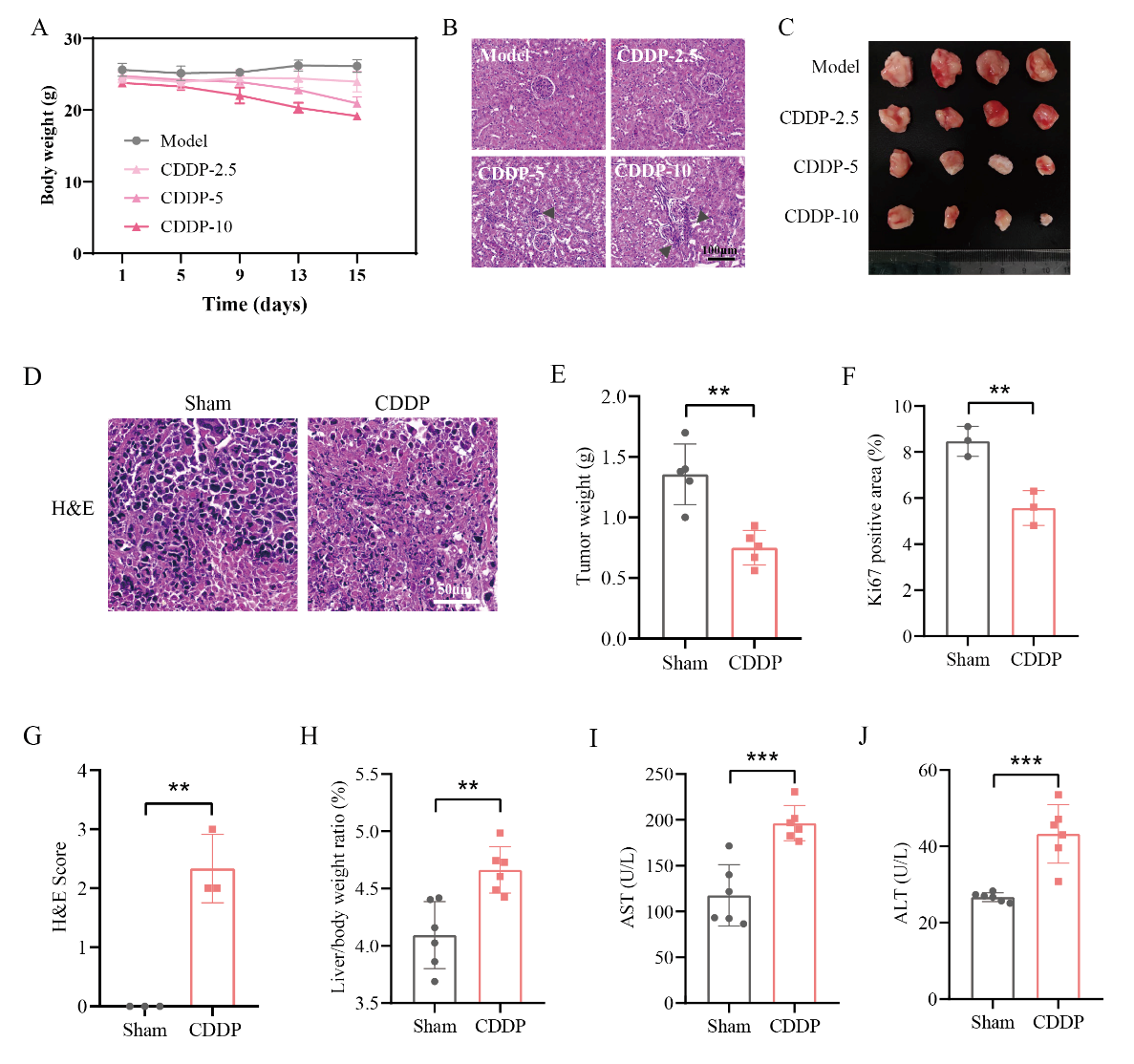
**

**Figure S1.** Cisplatin exerts chemotherapeutic effect and induces nephrotoxicity. (A) Body weight statistics in preliminary experiments. (B) H&E staining of kidney (scale bar = 100 μm) in preliminary experiments. (C) Representative images of tumors isolated from Model and CDDP groups mice in preliminary experiments. (D) H&E staining of tumor in Sham and CDDP treatment group (scale bar = 50 μm). (E) Tumor weight in two group (n = 5). (F) Immunohistochemical findings for Ki67 marker positive area ratio corresponding to be Figure 1D (n = 3). (G) Renal pathological score statistics corresponding to be Figure 1F (n = 3). (H) The liver/body weight ratio in two group (n = 6). (I-J) Effects of CDDP on the levels of serum ALT and AST in two group (n = 6). The error bars indicate the means ± SD. The *p* values were determined by Student’s t test; ***p* < 0.01, ****p* < 0.001 *vs*. Sham.

**
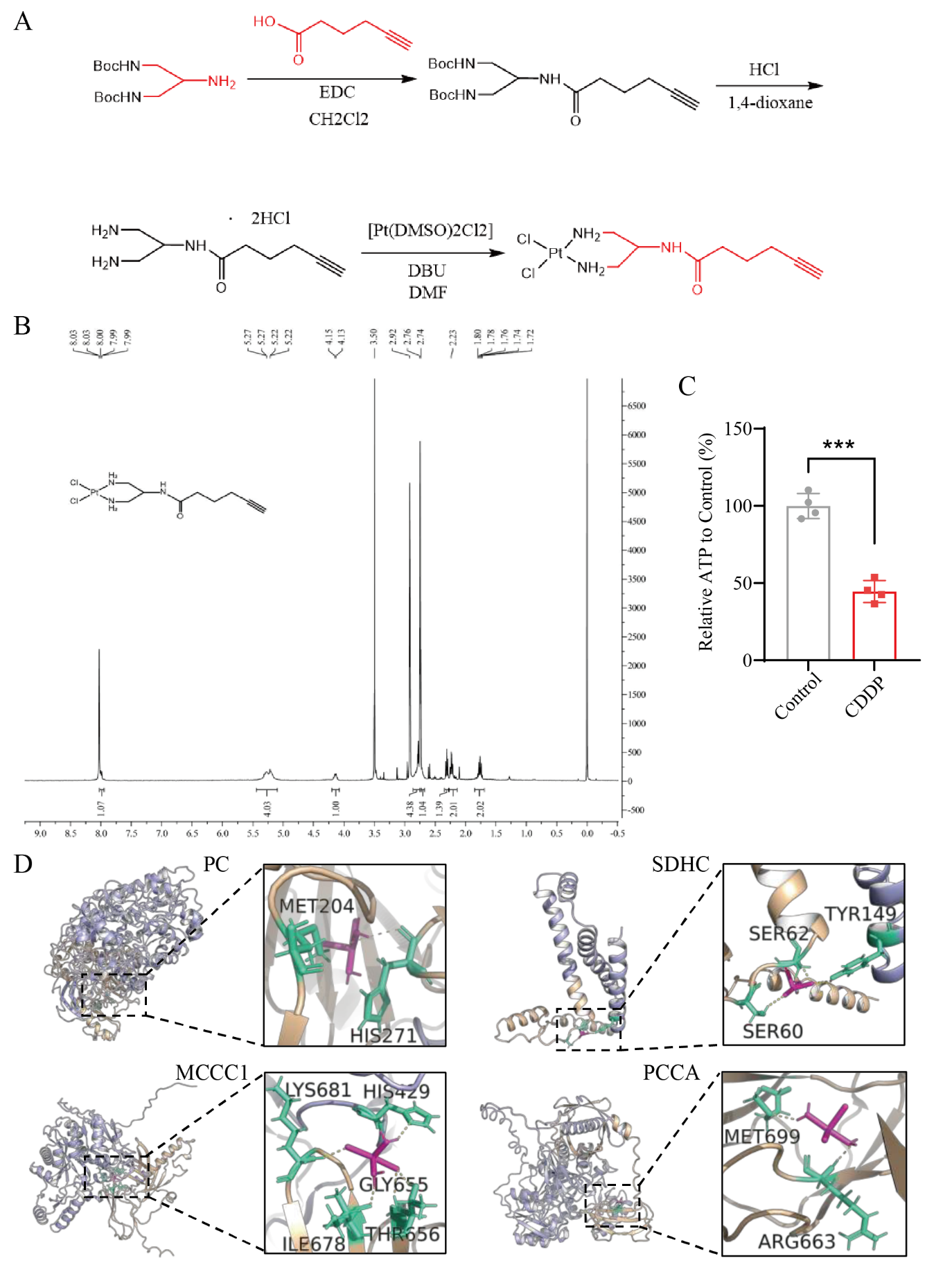
**

**Figure S2**. The synthesis route and H^1^NMR spectrum of the cisplatin probe. (A-B) The　CDDP probe (CDDP-P) was designed and synthesized as previously reported[[1](#_ENREF_1),[2](#_ENREF_2)]. ^1^H NMR of CDDP-P ^1^H NMR (400 MHz, DMF-d7): δ 8.02 – 7.95 (m, 1H), 5.44 – 5.09 (m, 4H), 4.14 (d, J = 7.7 Hz, 1H), 2.86 – 2.75 (m, 4H), 2.72 (m, 1H), 2.31 (t, J = 7.5 Hz, 2H), 2.23 (td, J = 7.1, 2.6 Hz, 2H), 1.76 (dt, J = 14.4, 7.2 Hz, 2H). (C) the effect of CDDP on ATP production in HK2 cells (n = 4). The error bars indicate the means ± SD. The *p* values were determined by Student’s t test; ****p* < 0.001 *vs.* Control. (D) Molecular docking between CDDP and target proteins.


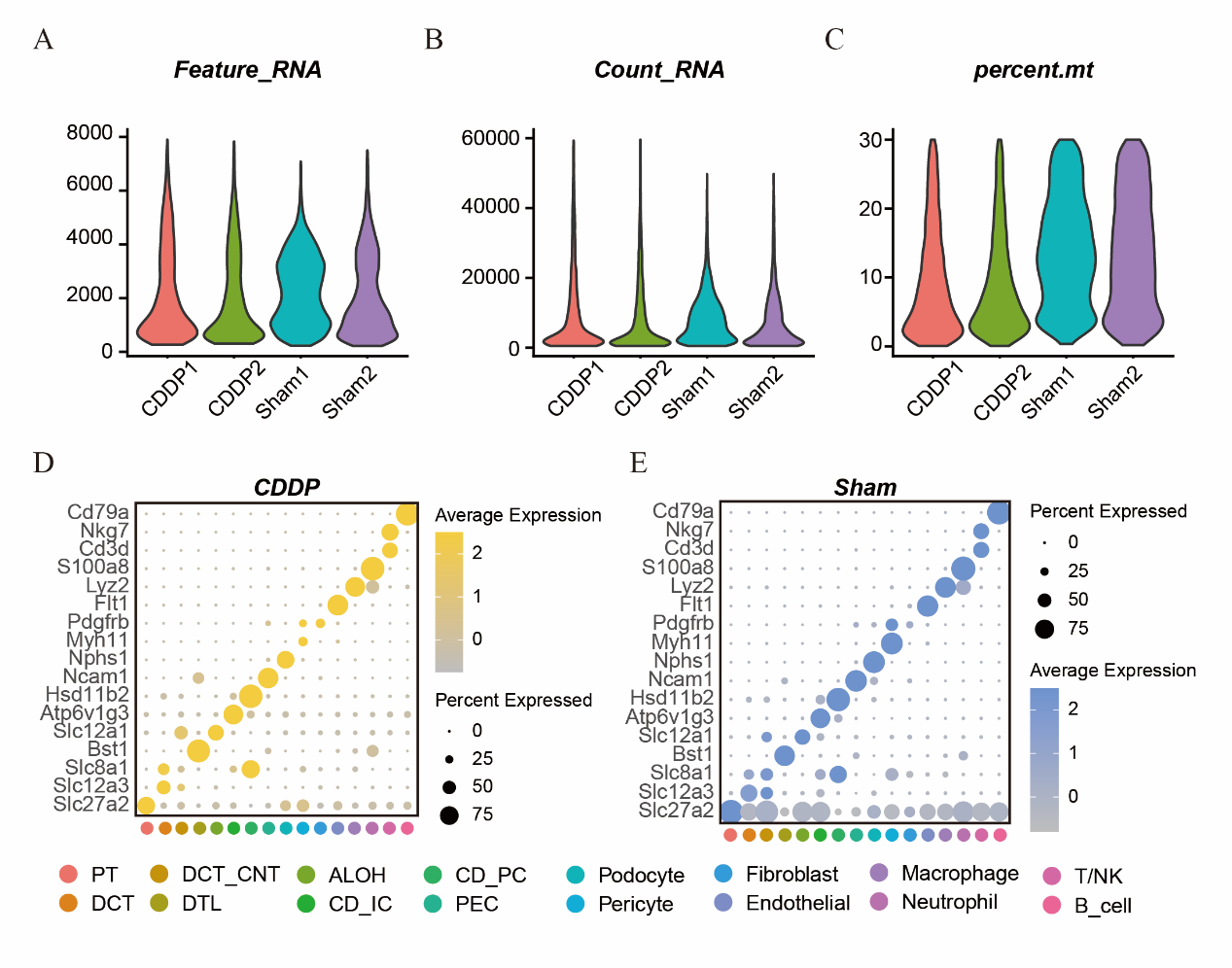


**Figure S3**. Quality control of scRNA data, marker genes distribution and cellular composition. (A-C) The number of genes (top), count of transcripts (middle) and percentage of mitochondrial genes (bottom) in each sample and group following quality control. (D-E) Dot plot shows the top marker genes of each cell types in CDDP and Sham group.


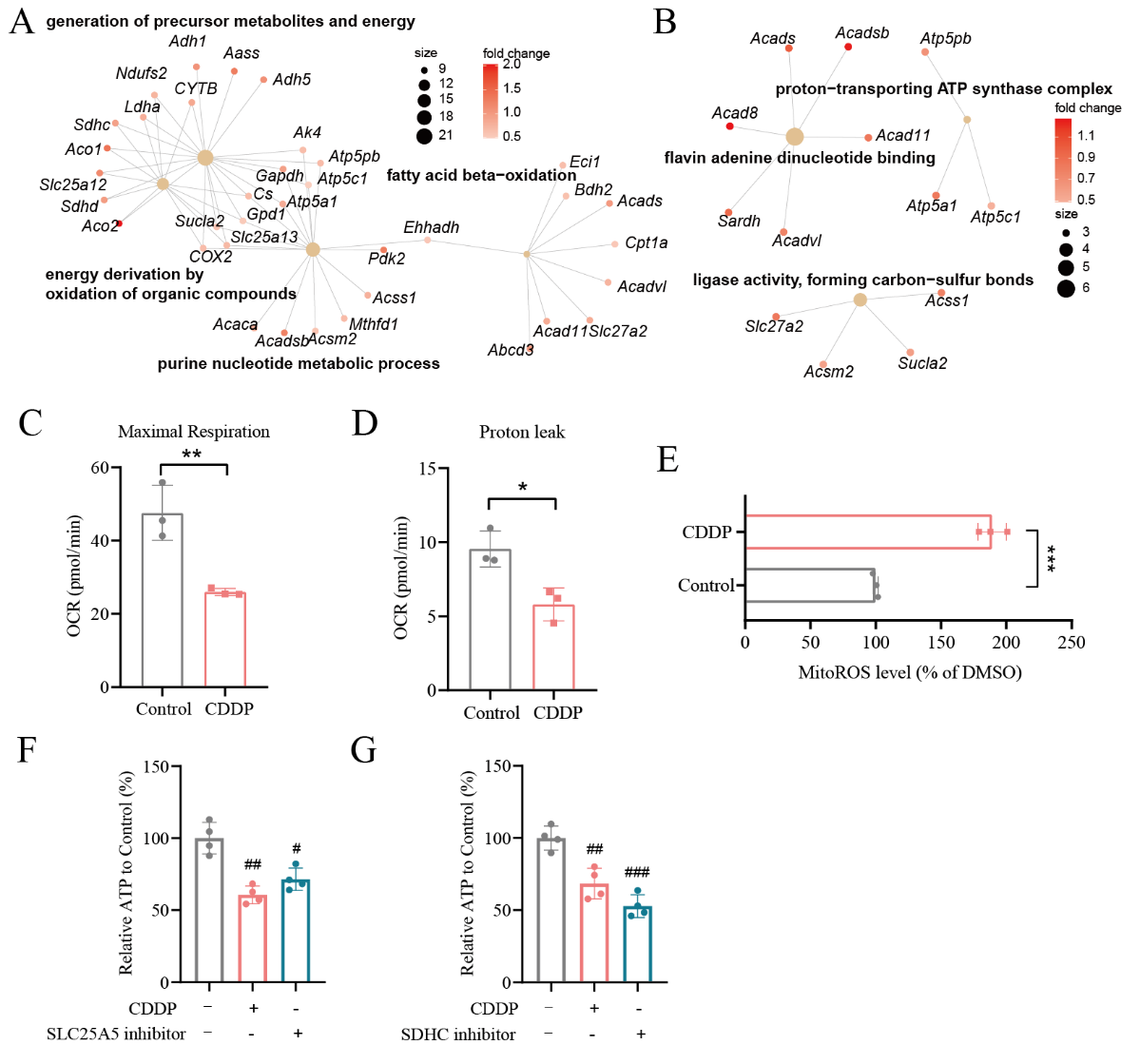


**Figure S4**. The biological functions of kidney and HK2 treated with CDDP. (A-B) An illustration of the metabolic signaling pathways enriched by these targeted proteins. (C) Maximal respiration capacity of OCR is examined in both groups (n = 3). (D) Proton leak capacity of OCR is examined in both groups (n = 3). (E) Statistics of flow cytometry detecting MitoROS corresponding to be Figure 4K (n = 3). The error bars indicate the means ± SD. The *p* values were determined by Student’s t test; **p* < 0.05, ***p* < 0.01, ****p* < 0.001 *vs*. Control). (F) Detection of ATP level in HK-2 cells treated with vehicle, CDDP, SLC25A5 inhibitor (Benzophenone) (n = 4). (G) Detection of ATP level in HK-2 cells treated with vehicle, CDDP, SDHC inhibitor (Atpenin A5) (n = 4). The error bars indicate the means ± SD. The p values were determined by One-way ANOVA; *#p* < 0.05, *##p* < 0.01, *###p* < 0.001 vs. Control.


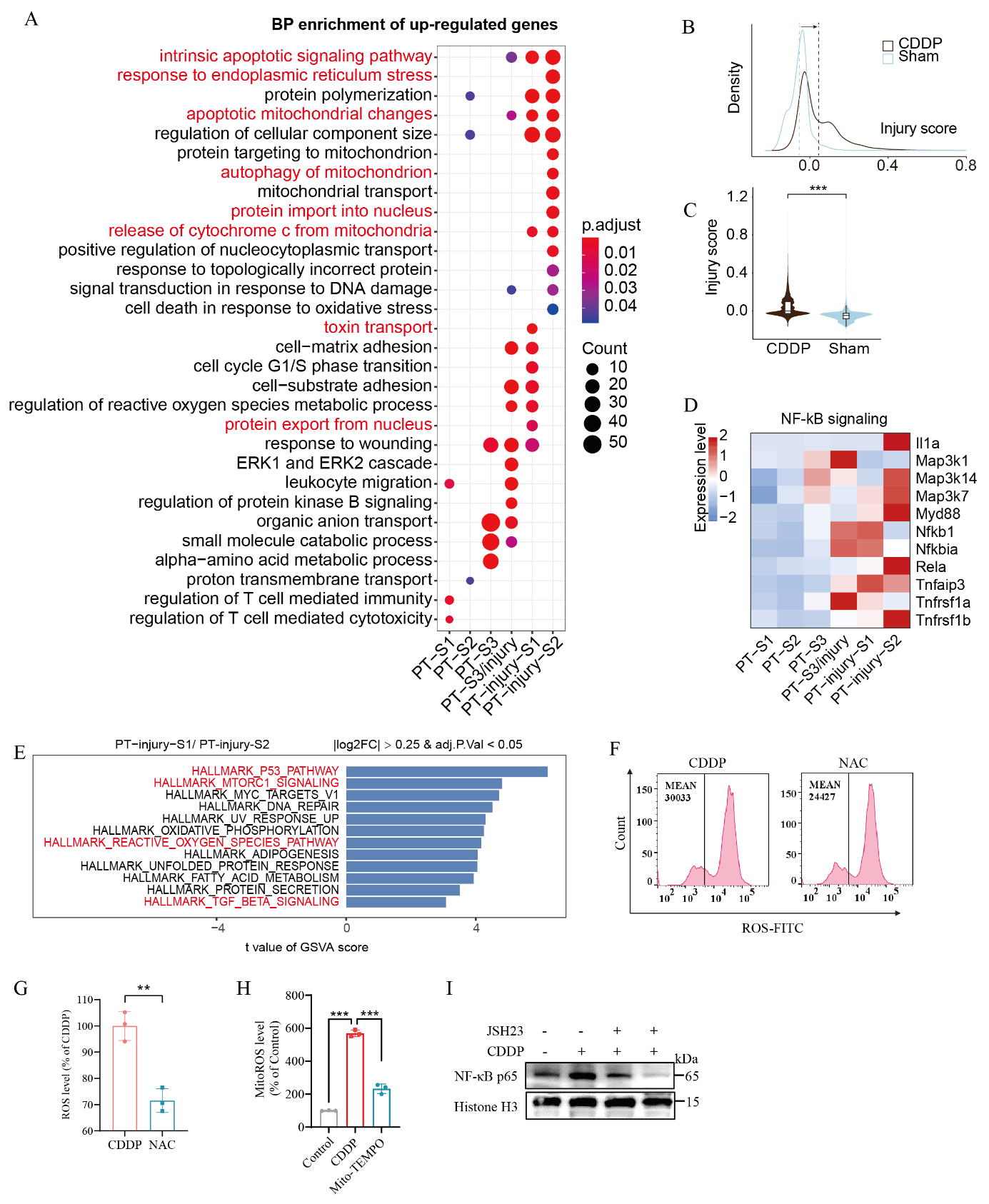


**Figure S5**. Cisplatin specifically induced proximal tubule (PT) cells injury via activating inflammatory response. (A) Functional profiling revealed distinct signatures in these PT cells. (B-C) The damage changes with injury scores in CDDP and Sham groups. (D) The expression changes in response to NF-kB signal among the injury-related PT cells. (E) GSVA shows inflammatory response and ROS signaling. (F-G) Flow cytometry to ROS levels of HK-2 cells treated with vehicle and CDDP (n=3). The error bars indicate the means ± SD. The *p* values were determined by Student’s t test; ***p* < 0.01 vs. Control. (H) Flow cytometry to MitoROS levels of HK-2 cells treated with vehicle (saline), CDDP, CDDP + Mito-TEMPO (n=3). The error bars indicate the means ± SD. The *p* values were determined by One-way ANOVA;  ****p* < 0.001 *vs*. CDDP group. (I) Western blotting to demonstrate the effect of NF-κB p-p65 in HK-2 cells treated with vehicle (saline), CDDP, CDDP + JSH23.


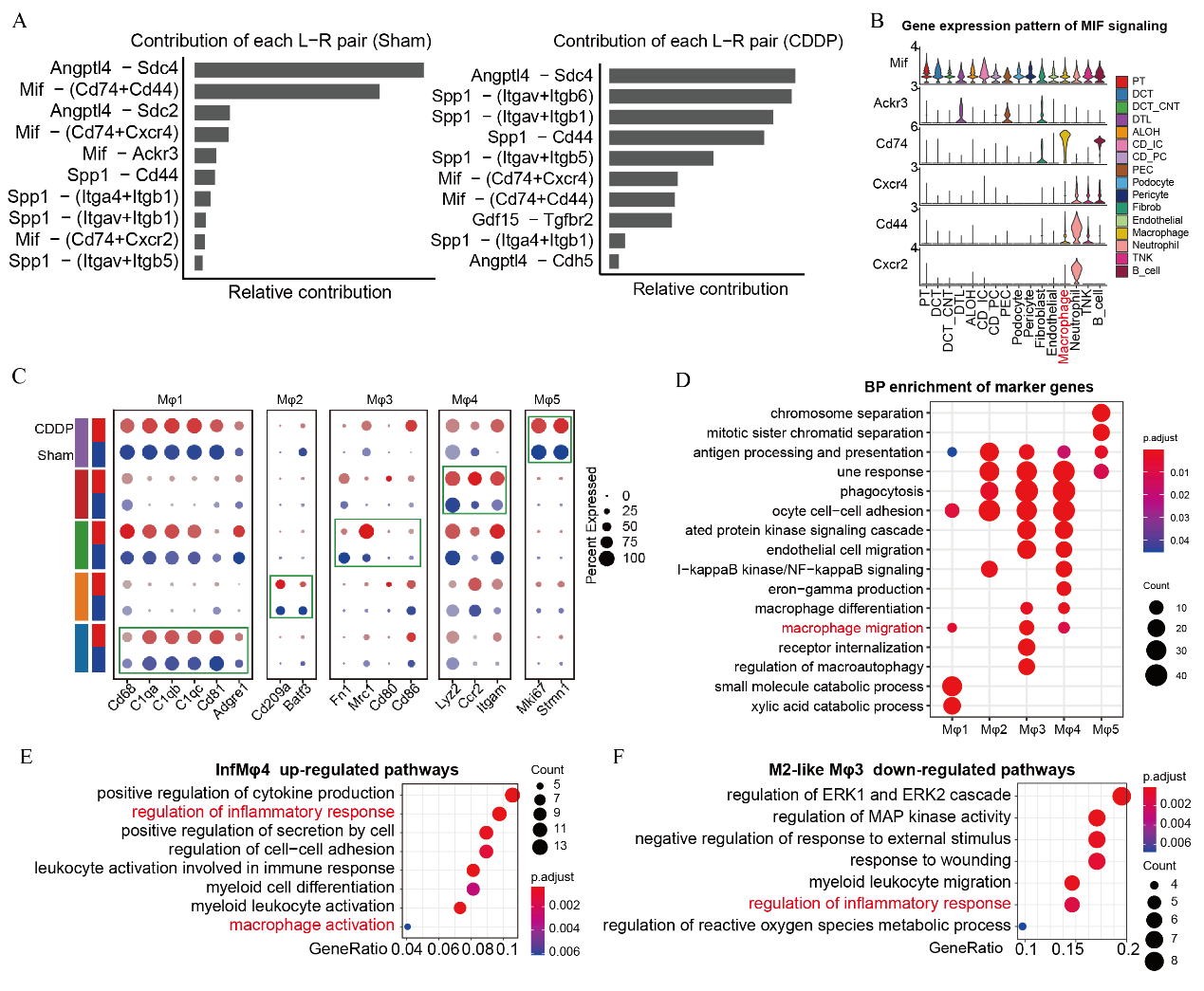


**Figure S6**. Cisplatin significantly induces renal infiltration and activation of macrophage. (A) Bar plot showing the relative contribution of each ligand-receptor pair to the overall MIF signaling network between sham group (left) and CDDP group (right). (B) Violin plot showing the expression patterns of signaling genes involved in the inferred MIF signaling network. (C) Dot plot shows the marker genes in immune cells. (D-F) GO analysis for the two infiltration subclusters for pro-inflammatory signaling and inflammation-related responses.


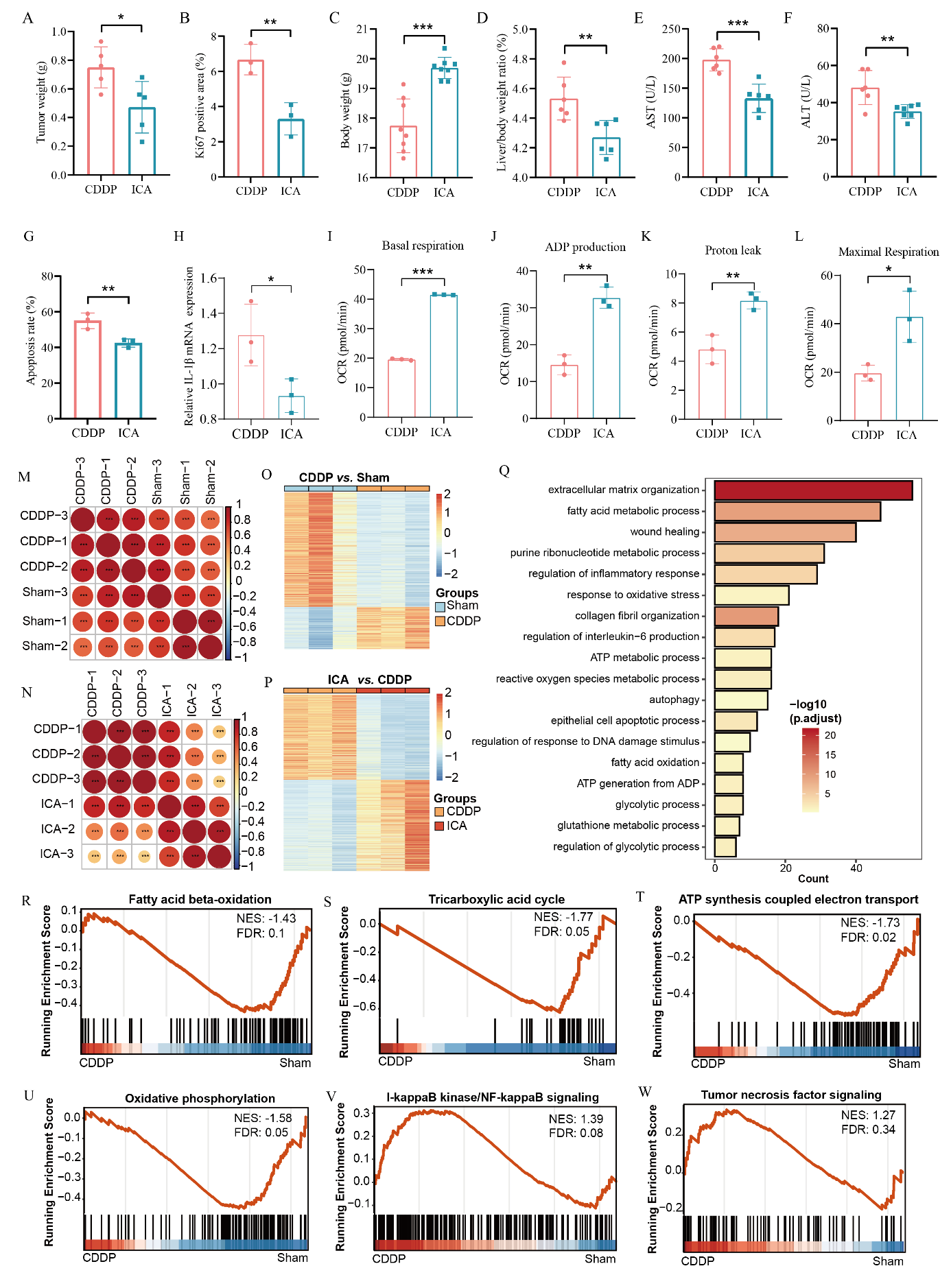


**Figure S7.** ICA in combination with CDDP, synergistically enhances anticancer efficacy while reducing the cisplatin-induced toxicity. (A) Tumor weight of tumor mice treated with alone CDDP or ICA in combination with CDDP: CDDP group and ICA group (n = 5). (B) Statistics for Ki67 marker positive area ratio corresponding to be Figure 1D (n = 3). (C) Body weight statistics in both groups (n = 8). (D) The liver/body weight ratio in both groups (n = 6). (E-F) Effects of CDDP on the levels of serum ALT and AST in two group (n = 6). (G) Statistics for apoptotic HK-2 corresponding to be Figure 7J (n = 3). (H) QPCR assay to examine effects of ICA on IL-1β in HK-2 cells treated with CDDP (n = 3). (I) Basal respiration, (J) ADP production, (K) proton leak, (L) maximal respiration capacity of OCR is examined in both groups (n = 3). (M-N) Correlation of samples in Sham, CDDP and ICA groups. (O-P) Expression patterns of DEGs in CDDP *vs.* Sham and ICA *vs.* CDDP. (Q) Biological process enrichment of DEGs in CDDP *vs.* Sham. (R-W) Pathway analysis of fatty acid β-oxidation, tricarboxylic acid cycle, ATP synthesis coupled electron transport, oxidative phosphorylation, NF-κB signaling and tumor necrosis factor pathway. The error bars indicate the means ± SD. The *p* values were determined by Student’s t test; **p* < 0.05, ***p* < 0.01, ****p* < 0.001 *vs*. CDDP group.


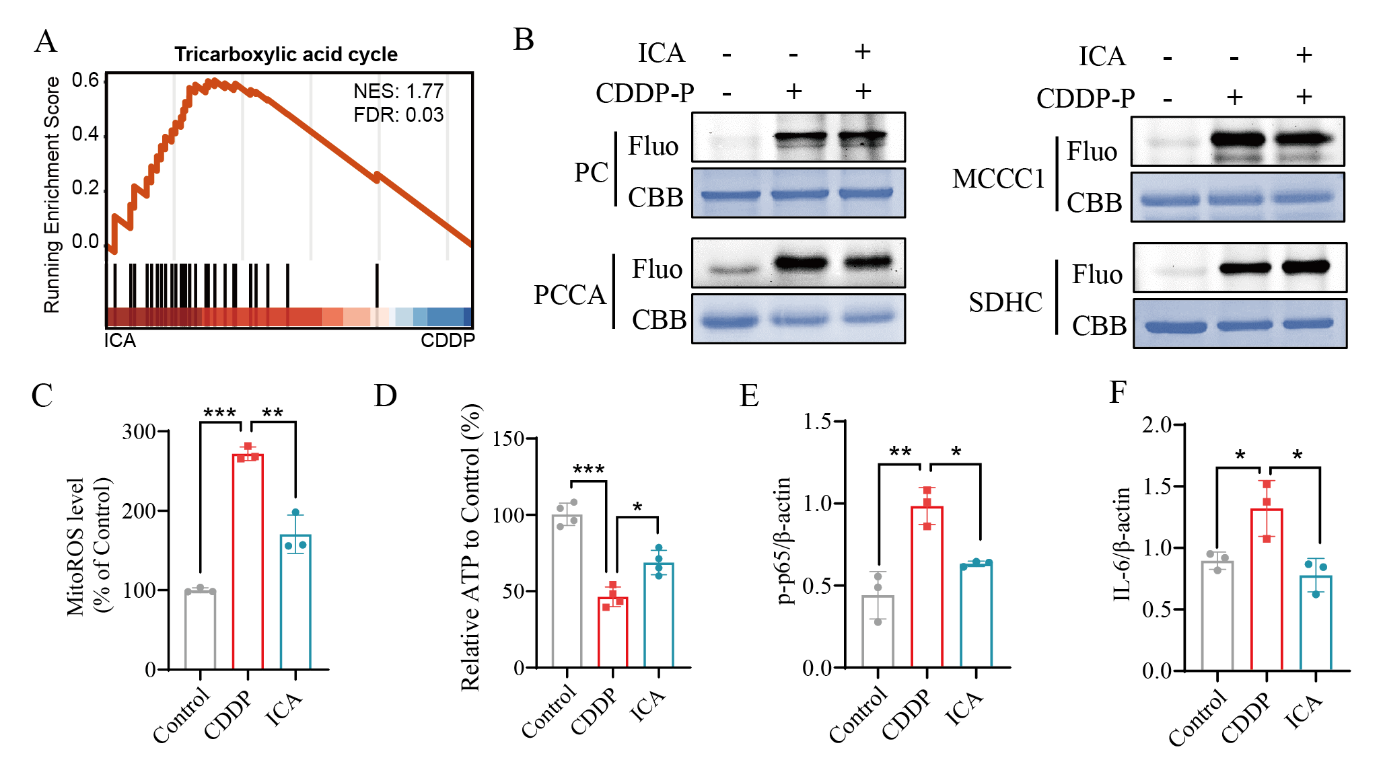


**Figure S8**. ICA ameliorates cisplatin-induced toxicity for mitochondrial metabolic and NF-κB-mediated inflammatory. (A) Gene sets analysis of tricarboxylic acid cycle. (B) Fluorescence labeling of recombinant protein domains to detect the effect of ICA on CDDP-bound target proteins. (C) Flow cytometry to MitoROS levels in HK-2 cells treated with vehicle, CDDP, CDDP + ICA (n = 3). (D) The effect of ICA on CDDP-inhibited ATP production in HK2 cells (n=3). (E-F) Western blotting to detect the phosphorylation of NF-κB and IL-6 in HK-2 cells treated with vehicle, CDDP, CDDP + ICA (n = 3). The error bars indicate the means ± SD. The *p* values were determined by One-way ANOVA; **p* < 0.05, ***p* < 0.01, ****p* < 0.001 *vs.* CDDP group.


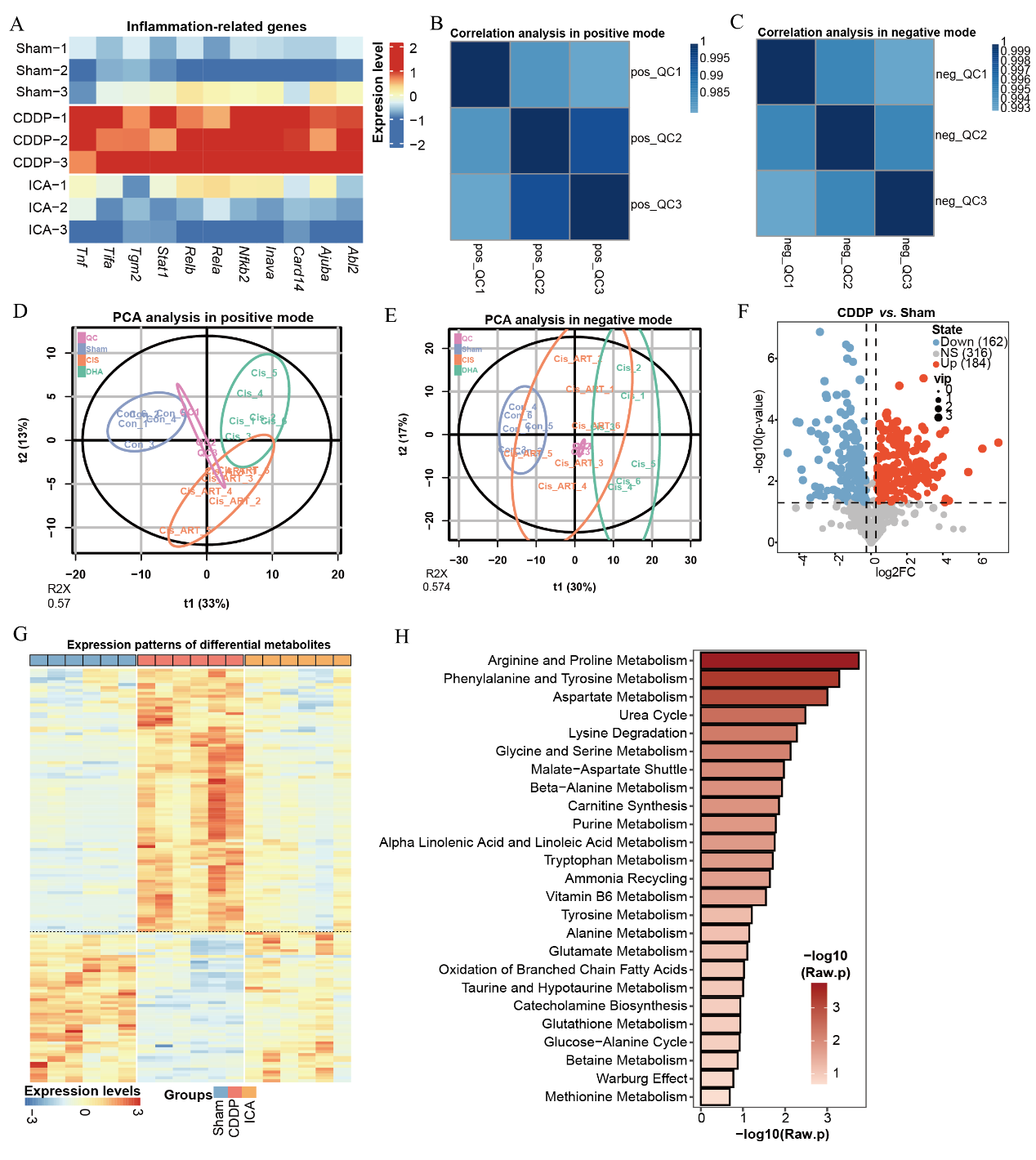


**Figure S9**. The metabolome reveals the mechanism of ICA alleviating CDDP-induced nephrotoxicity. (A) Expression patterns of inflammatory genes in Sham, CDDP and ICA group (B-C) Relationship of quality control (QC) samples in positive and negative modes. (D-E) PCA analysis of samples in QC, Sham, CDDP and ICA group in both modes. (F) Volcano plot shows contents distribution of DEMs. (G) Expression patterns of DEMs in Sham, CDDP and ICA group. (H) KEGG enrichment of DEMs in CDDP *vs.* Sham.

**Table S1**. gene sequence

| Gene name | FP： | RP： |
| --- | --- | --- |
| β-actin | CATGTACGTTGCTATCCAGGC | CTCCTTAATGTCACGCACGAT |
| IL-6 | ACTCACCTCTTCAGAACGAATTG | CCATCTTTGGAAGGTTCAGGTTG |
| IL-1β | ATGATGGCTTATTACAGTGGCAA | GTCGGAGATTCGTAGCTGGA |

**Reference**

1. Chen J, Chu Z, Zhang Q, Wang C, Luo P, Zhang Y*, et al.* STEP: profiling cellular-specific targets and pathways of bioactive small molecules in tissues via integrating single-cell transcriptomics and chemoproteomics. Chem Sci. 2024;15(12):4313-21.

2. White JD, Guzman LE, Zakharov LN, Haley MM, Derose VJ. An alkyne-appended, click-ready Pt(II) complex with an unusual arrangement in the solid state. Angew Chem Int Ed Engl. 2015;54(3):1032-5.
